# Non-invasive pharmacological advances in early retinopathy treatment: bioactive herbal compounds, polymer delivery systems, and computational bioprospecting of functional targets

**DOI:** 10.1007/s43440-025-00778-7

**Published:** 2025-08-28

**Authors:** Christopher Busayo Olowosoke, Thilini Thrimawithana, Tien Huynh

**Affiliations:** 1 School of Science, RMIT University, PO Box 71, Bundoora, VIC 3083 Australia; 2 School of Health and Biomedical Sciences, RMIT University, PO Box 71, Bundoora, VIC 3083 Australia; 3 Department of Biotechnology, School of Life Sciences (SLS), Federal University of Technology, Akure, Ondo State, PMB 704 Nigeria

**Keywords:** Herbal-based compounds, Macular carotenoids, Medtech, Wearable medicines, Computational methodology, Retinopathy, Polymers

## Abstract

Anti-vascular endothelial growth factor (anti-VEGF), laser, and vitrectomy therapy are commonly used for the management of vision-threatening posterior eye disease (PED), but non-invasive alternatives have garnered increasing popularity as proactive preventative strategies for early retinopathy, such as targeted plant-based diets or herbal supplements. However, only some plants contain bioactive compounds that specifically target retinal degeneration and demonstrate potent pharmacological benefits that are cost-effective, safe, and accessible for at-risk individuals. This review pinpoints plant bioactive compounds, specifically polyphenols and carotenoids, that target retinopathy, with a focus on apoptotic, angiogenesis, inflammation, and oxidative pathways leading to visible, functional, and vascular macula improvements. Innovations accelerating therapeutic applications of these botanicals for ocular delivery were then explored. Finally, advancements in disease assessments and the computational methods for early retinopathy biomarker diagnosis and treatment, particularly designed to bio-prospect plant-based therapies, were also reviewed to guide future developments and address translational limitations.

## Abstract (significance)

Clinical trial number

Not applicable.

## Key summary points

• Retinopathy is a secondary damage of retinal neurons and vascular cells caused by metabolic/cardiovascular disease or radiation exposure, leading to vision impairment or blindness.
• Specific bioactive compounds from plant sources have potential as a long-term preventative strategy for early-stage retinopathy.
• These botanicals are polyphenols and carotenoids that are unstable and require technological innovations for targeted ocular applications.
• To facilitate ocular applications, polymers like hydrogels are suitable for bioprinting wearable ocular devices like contact lenses, implants, and inserts that can advance non-invasive delivery of bioactive compounds directly to the eyes.
• Computational and AI-assisted methods are innovative approaches for bioprospecting bioactive compounds and protein targets specific to retinopathy pathways to reduce costs on experimental resources and time and fast-track clinical validation for drug development.

## Graphical abstract

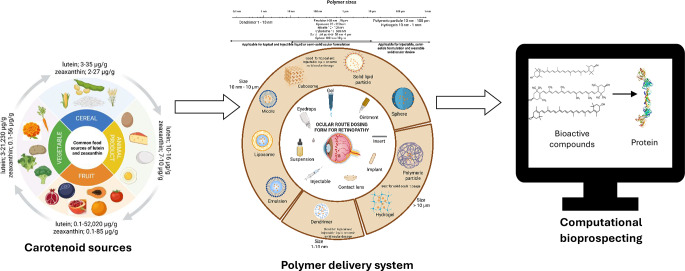

## Introduction

Retinopathy is a disease characterised by progressive damage to the retinal microvasculature and neural tissue, affecting over 103 million individuals globally as of 2020, and leading to retinal degeneration and vision loss [1]. Promising research has highlighted the potential of plant-derived bioactive compounds, specifically polyphenols and carotenoids, as preventative strategies for retinopathy. These natural compounds are not only cost-effective and safe but also accessible, making them particularly valuable for at-risk populations where conventional therapies may be limited [2].

This review aimed to provide a critical analysis of the evidence available for the use of polyphenols and carotenoids in the prevention of retinopathy due to their antioxidant, anti-inflammatory, anti-apoptotic, and anti-angiogenesis properties. The current reliance on oral supplementation is limited by poor bioavailability and inefficient delivery to retinal tissues, which significantly reduces their therapeutic efficacy. As a result, there is a pressing need to identify superior plant sources and develop advanced delivery methods beyond oral administration to maximize the protective benefits of the plant compounds for retinopathy [3]. Therefore, this review will also provide a comprehensive analysis of the polymers used to encapsulate plant bioactive compounds so they can be more efficiently delivered to the target tissue.

Finally, computational methods were evaluated to assess current evidence supporting plant-based preventative strategies for retinopathy, with a focus on bioprospecting for clinical translation. These recent advances in this field highlighted the promise of phytochemicals as accessible protective interventions for reducing the global burden of retinopathy.

## Methodology for literature review

This literature review aimed to synthesise evidence on bioactive herbal products, polyphenols, and carotenoids, for retinopathy management and prevention. The review addressed the following key research questions: (1) which plant-derived bioactive compounds show therapeutics potential for retinopathy management; (2) what polymer systems are used in the fabrication of ocular inserts for non-invasive delivery of therapeutics; (3) how are computational and AI-assisted methods used to improve biomarker diagnosis and bioprospecting for retinopathy treatments in this context?

A comprehensive search was conducted in PubMed, Google Scholar, Scopus, and Web of Science for peer-reviewed English language articles published between 1990 and 2025. The search terms included: “retinopathy”, “bioactive compounds”, “natural products”, “computational drug discovery”, “bioprospecting”, “polymer delivery systems”, and “non-invasive treatment”. Specific exclusion criteria were articles published in a language other than English, studies without a focus on polyphenols or macular carotenoids, and studies that did not focus on retinopathy. Titles and abstracts of all relevant articles were initially screened before the full text review. The review grouped studies that answered the three research questions above.

## Bioactive compounds commonly used for early retinopathy management

There is compelling evidence from cellular studies and animal trials that supports the efficacy of herbal therapy as an early intervention for retinopathy (Table 1). Notably, there was significant data from animal trials (85%) that demonstrated well-characterised mechanisms by which therapeutic interventions simultaneously target multiple interconnected pathological pathways in retinopathy, specifically apoptosis, aberrant angiogenesis, chronic inflammation, and oxidative stress (53%). The action of these plants was mainly by mitigation of inflammation (52%), angiogenesis (38%), oxidation (35%), and apoptosis (15%), and indicates the complex crosstalk in retinopathy progression.

**Table 1:** Plant species that have potential for early retinopathy prevention from cellular studies and animal trials, acting against angiogenesis, apoptosis, inflammation, and oxidative stress

Plant species	Parts used	Extraction	Phytochemical	Model	Mechanism of action	References
*Allium sativum* (garlic)	Fresh bulb	250 mL water at 20–25 °C, time not specified	Whole extract	Male Wistar rat	↓ total oxidants, lipid peroxidation, TGF-β2 + IL-1β. ↑ antioxidant ability + + thiol.	[4]
*Andrographis paniculate* (king of bitter)	Dried leaf	96% ethanol for 72h, temperature not specified	Whole extract	Male Wistar rat	↑ GSH, SOD, + CAT. ↓ VEGF + TNFα. ↓ vessel diameter	[5]
*Arctium lappa* (burdock)	Dried fruit	95% ethanol at 80 °C for 3h, defatted with petroleum ether at 60–90 °C, final extract in ethyl acetate	Whole extract has lignan, matairesinol, arctigenin, lappaol A, lappaol F, lappaol C, arctignan E, arctiin, lappaol H	Male Wistar rat	↓ VEGF, PKCβ2. Prevented leakage by 50%	[6]
*Aster koraiensis* (Korean aster)	Flower, leaf, stem	100% ethanol at 20–25 °C for 3d	Whole extract has chlorogenic acid, 3,5-di-O-caffeoylquinic acid	Male Sprague-Dawley rat	↓ AGE + RAGE binding activity. ↓ injuries to retinal vascular cells.	[7]
*Astragalus mongholicus* (Mongolia milkvetch)	Dried root	10mL 75% methanol mixed for 0.17h, temperature not specified	Whole extract	Male C57BL/KsJ-db/db + C57BL/KsJ-db/m mice	Regulated phenylalanine metabolism, steroid hormone biosynthesis, sphingolipid metabolism, AMPK, ERK1/2, PI3K, + p70 S6K.	[8]
*Pseudostellaria heterophylla* (false starwort)	Dried root tuber	10 mL 75% methanol mixed for 0.17h, temperature not specified	Whole extract	Male C57BL/KsJ-db/db + C57BL/KsJ-db/m mice		
*Ligustrum lucidum* (glossy privet)	Dried mature fruit	10 mL 75% methanol mixed for 0.17h, temperature not specified	Whole extract	Male C57BL/KsJ-db/db + C57BL/KsJ-db/m mice		
*Lycium barbarum* (wolfberry)	Dried mature fruit	10 mL 75% methanol mixed for 0.17h, temperature not specified	Whole extract	Male C57BL/KsJ-db/db + C57BL/KsJ-db/m mice		
*Rheum officinale* (Chinese rhubarb)	Dried root and rhizome	10 mL 75% methanol mixed for 0.17h, temperature not specified	Whole extract	Male C57BL/KsJ-db/db + C57BL/KsJ-db/m mice		
*Cichorium intybus* (chicory)	Root	250 mL 80% methanol at 50–60 °C for 5–6h	Kaempferol, apigenin, pelargonidin	Red blood cell	↓ ALR, AGE, + sorbitol accumulation in RBCs	[9]
*Chrysanthemum morifolum* (garden mum)	Flowers	2000 mL distilled water at 120 °C for 0.75h	Whole extract	Male Sprague-Dawley rat	No deflection to a-wave + b-wave amplitude. No morphological changes to retina layers.	[10]
*Lycium barbarum* (wolfberry)	Dried fruit	2000 mL distilled water at 120 °C for 0.75h	Whole extract	Male Sprague-Dawley rat		
*Dendrobium chrysotoxum* (fried-egg orchid)	Dried whole plant (fruit, leaf, root, stem, flower)	10 L of 75% ethanol heated for 3h, temperature not specified	Whole extract has erianin, gigantol, and oscatilin	Sprague-Dawley rat (unspecified sex)	↓ MMP2/9, IL-1β, IL-6, ICAM-1, phosphorylation of p65, PDGF, bFGF, IGF-1, VEGF/VEGFR2.	[11]
*Euterpe oleracea* (acai palm)	Pulp	Ultrapure aqueous medium, warm at 60 °C for 3h	Whole extract	Swiss mice of both sexes	↓ amplitude of the visual electrophysiological response.	[12]
*Herba scutellaria barbata* (barb skullcap)	Dried whole plant (fruit, flower, root, stem)	80% ethanol at 20–25 °C for 2h	Whole extract	Male C57BL/6J mice	↑ claudin-1 + claudin-19. ↓ TNF-α, IL-1β, ICAM-1 + NFκB p65. Nullify microglia cell activation.	[13]
*Juglans regia* (common walnut)	Dried leaf	Methanol at 20–25 °C for 72h	Whole extract	Male Sprague-Dawley rats	↓ caspase-3, COX-2, PARP, + S100B.	[14]
*Litchi chinensis* (lychee)	Fruit pericarp powder	2% gum acacia, time not specified, temperature not specified	Whole extract	Male albino Wistar rat	↓ AGE, ALR, glucose level, + PCO. ↑ SOD, CAT, + GSH.	[15]
*Lonicera japonica* (Japanese honeysuckle)	Dried flower bud	1000 mL of distilled water heated for 4h, temperature not specified	Whole extract has chlorogenic acid, caffeic acid, and luteolin	C57BL/6 mice (unspecified sex), RF/6A cells	↓ VEGF-induced RF/6A cell proliferation. ↓ retinal angiogenesis.	[16]
*Lycium barbarum* (wolfberry)	Dried fruit powder	2.5 mL methanol at 20–25 °C for 15 min, then 100% methanol + water 1:1 at room temperature 20–25 °C for 15 min	Whole extract has taurine	ARPE-19 cells	↓ pro-inflammatory mediators encoding MMP9, COX-2, iNOS + fibronectin. ↑ PPAR-γ.	[17]
*Morus alba* (white mulberry)	Dried lead powder	90% ethanol for 72h at ambient temperature	Whole extract	Male Wistar rat	↓ VEGF, caspase-3, Bax, TNFα, IL1β. ↑ Bcl-2, ↑ SOD, CAT, GPx, + GSH. ↓ MDA, + PKCβ.	[18]
*Mangifera indica* (mango)	Seed	50% hydroalcoholic at 20–25 °C for 3d. (1 volume of extract combined with the other extract)	Whole extract has quercetin, gallic acid	Male Wistar rat	↓ ALR, p38MAPK, ERK1/2, VEGF.	[19]
*Polygonum odoratum* (Vietnamese coriander)	Aerial part (leaf, stem, flower)	50% hydroalcoholic at 20–25 °C for 3d. (5 volume of extract combined with the other extract)	Whole extract has quercetin, gallic acid	Male Wistar rat		
*Magnolia officinalis* (hou po)	Dried root bark	80% ethanol + distil water at 20–25 °C for 1w	Magnolol	ARPE-19 cells	↓ TGF-β1 + fibronectin expression, MAPK/Akt, ERK.	[20]
*Momordica cochinchinensis* (gac fruit)	Freeze-dried peel, pulp, seed aril	20mL 70% ethanol for 2h, temperature not specified	Whole extract	ARPE-19 cells	↓ ROS + VEGF. ↑ PEDF.	[21]
*Moringa oleifera* (moringa)	Dried leaf	Water at 60–70 °C, time not specified	Whole extract has quercetin, gallic acid	Albino Wistar rat of both sexes	↓ TNF-α, + IL-1β. Prevent thickening of the capillary basement membrane.	[22]
*Ocimum sanctum* (holy basil)	Dried leaf	Distilled water at 20–25 °C for 24h	Whole extract	Male albino Wistar rat	↓ lipid peroxidation, HbA1c plasma + glucose. ↑ GPx, SOD, CAT + GST.	[23]
*Panax quinquefolius* (American ginseng)	Dried root	75/25 v/v; ethanol/water at 40 °C for 5h	Ginsenoside Rg1, ginsenoside Re, ginsenoside Rb1, ginsenoside Rc, ginsenosideRb2	Male C57BL/6 mice, Male db/db mice	↓ VEGF, ET-1, + TGF-β1.	[24]
*Polygonum cuspidatum* (Japanese knotweed)	Dried root	100% ethanol, 20–25 °C for 3d	Whole extract has resveratrol-3-O-β-D-glucopyranoside (polydatin), resveratrol, emodin-5-O-β-D-glucopyranoside, emodin	Male Sprague-Dawley rat	↓ HMGB1, + RAGE expression. ↓ NF-κB activity, NF-κB binding to RAGE promoter.	[25]
*Spinacia oleracea* (Spinach)	Dried leaf	Methanol at 20–25 °C, time not specified	Whole extract	Male Wistar rat	↓ CML-RAGE co-localization, NOX4, iNOS, NT, + MDA. ↓ VEGF, NF-κB, S100B, GFAP. ↓ apoptosis.	[26]
*Tinospora cordifolia* (giloy)	Stem	Methanol soluble, concentration not specified, time not specified, and temperature not specified	Whole extract	Wistar rats of both sexes	↓ VEGF, IL-1β, TNF-α, + PKC ↓ blood glucose, retinal basement thickening. ↑ GSH, SOD, and CAT.	[27]
*Trigonella foenum-graceum* (fenugreek)	Dried seed powder	50 mL of Methanol for 1h, temperature not specified	Whole extract has saponins	Albino Wistar rat of both sexes	↓ TNF-α, IL-1β, PKC-β, + VEGF. No vascular leakage, thickening of the basement membrane.	[28]
*Ulmus davidiana* (David elm)	Branches (stem with bark)	60% ethanol at 20–25 °C, time not specified	Whole extract has catechin 7-O-b-D-apiofuranoside	HPP + HRMEC cells	↓ activation of p38, JNK, and TNF-α. ↓ pericyte apoptosis.	[29]
*Zingiber officinale* (ginger)	Dried root	95% ethanol for 24h, temperature not specified	Whole extract has 6-gingerol, 8-gingerol, 10-gingerol, 6-shogaol	Male Wistar rat	↓ VEGF, NF-κB, + TNF-α. ↑ eNOS + G6PDH.	[30]
*Zingiber zerumbet* (shampoo ginger)	Dried rhizome powder	10 L of 95% ethanol at 20–25 °C for 7d	Whole extract has kaempferol, curcumin, and zerumbone	Male Wistar rat	↓ VEGF, PEDF, IL-6, ICAM-1, NF-κB, p65, ERK1/2.	[31]

Abbreviations: Akt: protein kinase b, ALR: aldose reductase, AMPK: adenosine monophosphate-activated protein kinase, Bax: bcl-2 associated x-protein, Bcl2: b-cell lymphoma protein 2, CAT: catalase, CML-RAGE: nε-carboxymethyl-lysine-receptor for advance glycation endproducts, COX-2: cyclooxygenase-2, eNOS: endothelial nitric oxide synthase, ERK1/2: extracellular signal-regulated protein kinase1/2, ET-1: endothelin-1, G6PDH: glucose-6-phosphate dehydrogenase, GFAP: glial fibrillary acidic protein, GSH: glutathione, HMGB1: high mobility group box 1 protein, ICAM-1: intercellular adhesion molecule-1, IL-1β: interleukins 1 beta, iNOS: inducible nitric oxide synthase, MCP1: monocyte chemotactic protein-1, MDA: malondialdehyde, MMP-9: matrix metalloproteinase-9, NF-κB: nuclear factor-kappa b, NT: 3-nitrotyrosine, p38MAPK: p38 mitogen-activated protein kinases, p70 S6K protein 70 S6 kinase, PARP-1: poly-(adp)-ribose polymerase-1, PEDF: pigment epithelium-derived factor, PI3K: phosphatidylinositol-3-kinase, PKC/β2: protein kinase C/beta 2, RBC; red blood cells, ROS: reactive oxidative species, S100B: calcium binding protein b, SDH: sorbitol dehydrogenase, SOD: superoxide dismutase, STAT3: signal transducer and activator of transcription 3, temp: temperature, TGF-β1/β2: transforming growth factor-beta 1/beta 2, TNF-α: tumor necrosis factor alpha, VEGF/R2: vascular endothelial growth factor/receptor 2

↑: increase/upregulate, ↓: decrease/downregulate, min; minute, d; day, h; hour, w; week

Cell culture- ARPE-19: retinal pigment epithelial cell line, HPP: human placental pericytes, HRMECs: human retinal microvascular endothelial cells

These plants are from diverse origins, and a total of 33 species were highlighted (Table 1). Many plants are indigenous species with established vernacular names and local distribution, enhancing accessibility for at-risk communities where conventional treatments are limited or unavailable. Pinpointing these specific plant species and the parts used enables a more targeted investigation of the bioactive compounds responsible for their protective effects, thereby streamlining the discovery and development of early retinopathy interventions, strengthening the scientific justification for advancing towards human clinical trials.

Although these plant species are well known, the current preparation and processing methods are not optimised for efficient extraction of the bioactive compounds that have efficacy against retinopathy (Table 1). Most plant materials were dried (70%), and optimum extraction solvents were alcohol-based, including methanol (32%), which is unsuitable for therapeutic use due to its toxicity, therefore requiring additional processing to remove. Consequently, despite the scientific knowledge, there are significant barriers to translation, particularly for dietary or oral delivery routes of these important plant species.

These plant species host multiple bioactive compounds that can work synergistically to elicit multiple mechanisms of action. These bioactive compounds are categorised as alkaloids, non-polyphenols (saponins), phenylpropanoids, polyketides, and terpenoids [32], but the most effective for retinopathy management are polyphenols and carotenoids, which target the signature pathological hallmarks of retinopathy, with or without diabetic origin [2, 33] and were reviewed in further detail.

### Polyphenols

**Fig. 1:**
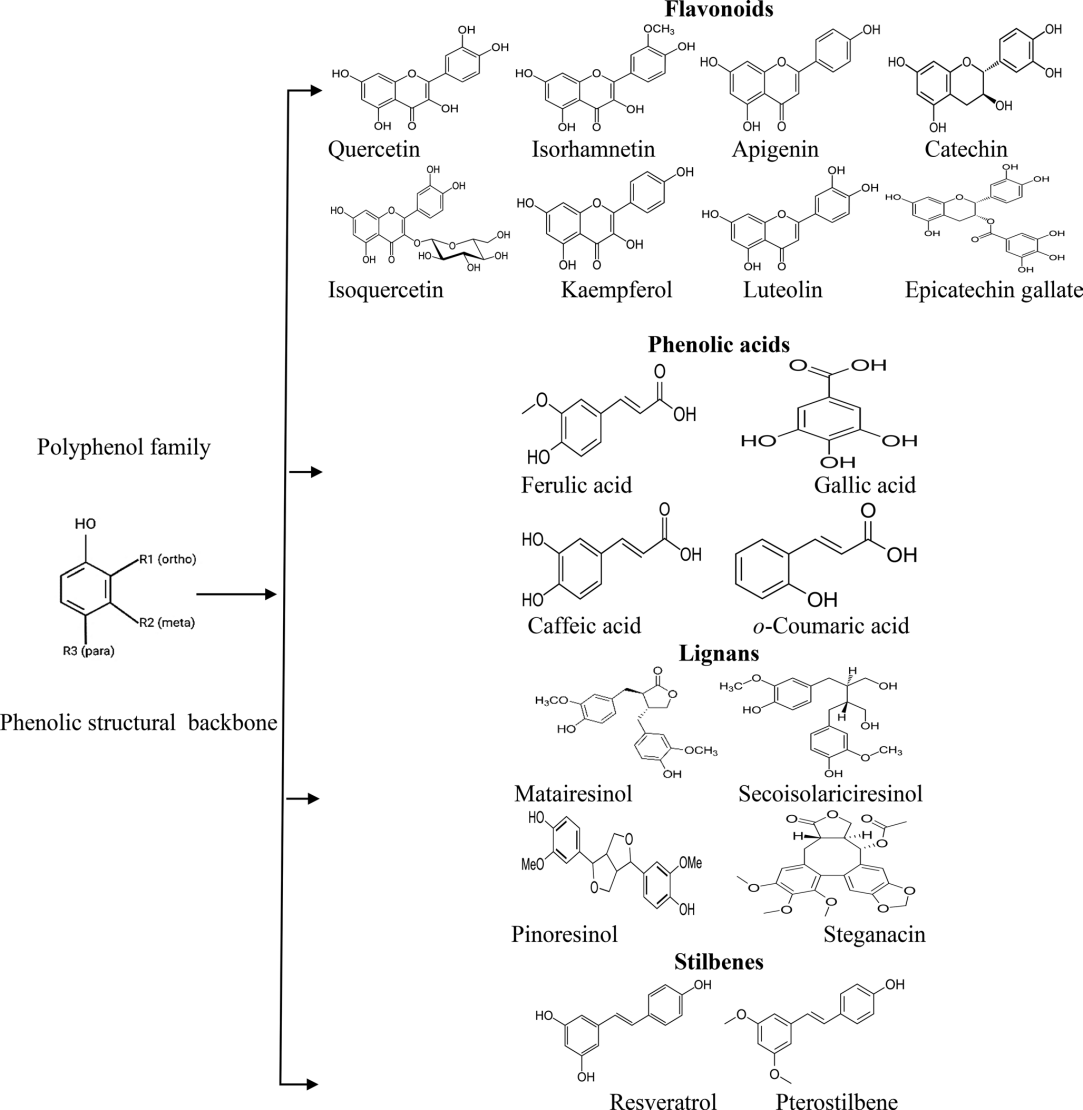
The phenolic backbone of the polyphenol family of natural compounds, the four subgroups, and their examples. R1, R2, and R3 are the side chains of the chemical structure that consists of different functional groups

Polyphenols are characterised by their phenolic structural backbone (Fig. 1). The polyphenol exists in four sub-groups: flavonoids, phenolic acids, lignans, and stilbenes. Flavonoids account for 60% of polyphenols, widely used as anti-cancer, antioxidation, antiviral, and antidiabetic agents [33].

These flavonoids can prevent permanent damage to the retina by suppressing proteins and receptors involved in oxidative stress, angiogenesis, apoptosis, and inflammation responses, typically in retinopathy and other ocular diseases [33, 34].

Generally, the high abundance of these polyphenols in common fruits and vegetables [35] makes it cost-effective and accessible in the tropics, where there is a greater probability of retinopathy occurrence due to high UV exposure, sickle cell disease, hypertension, and high consumption of diabetic prone diets, which can cause different forms of retinopathy. This makes polyphenols the ideal candidates for awareness and inclusion in dietary recommendations with practical approaches to mitigate the risk of retinopathy in the general population.

The therapeutic benefits of polyphenols in retinopathy are strongly supported by both cellular (Table 2) and animal trials (Table 3). These studies identified 9 key purified polyphenolic compounds that demonstrate significant protective effects by targeting key pathological processes against oxidation, angiogenesis, apoptosis, and inflammation [2, 33]. Specifically, polyphenols were potent inhibitors of proinflammatory cytokines and ROS that induce vascular damage [2]. The nuclear factor-kappa B (NF-κB) pathway has also been widely implicated, which suggests the contribution of cytokines and chemokines in retinal pathogenesis [36, 37]. Consequently, this reduces or reverses neovascularization, depletion of retinal layers, and vascular permeability, which are hallmarks of retinopathy. These polyphenols exert multiple benefits targeting several major pathological pathways associated with chronic diseases and provide a more targeted and multifaceted intervention compared to non-specific antioxidant or single target therapies. Notably, these isolated and purified polyphenols are already commercially available and show much promise [38] for adoption; however, their widespread recommendation by clinicians remains limited. This is primarily due to the absence of large-scale, well-controlled human clinical trials and the lack of standardized formulations [39].

**Table 2:** In-vitro studies of polyphenols for the treatment of early retinopathy

Polyphenol	Subclass of compound (%purity)	Natural Source	Mode of action classification	Dose*	Cell model/stressor	Outcome of polyphenol treatment	References
Anthocyanin	Cyanidin, cyanidin 3-glucoside, cyanidin 3,5-diglucoside	Elderberry	Anti-oxidation	**T**: 0.05, 0.1 + 0.5 mg/mL, **C**: H_2_O_2,_ AAPH + FeSO_4_/AA stress cell only.	BAEC + HAEC. **S**: 75–300 µM H_2_O_2,_ 8–32 µM AAPH + 2.5–250/0.15–1.5 µM FeSO_4_/AA for 2h.	T is beneficial. T penetrates the cytosol ↓H_2_O_2_, AAPH + FeSO_4_/AA effect on cell. ↓cytotoxicity by 20–40%	[40]
	Malvidin-3-glucoside (glc), malvidin-3-galactoside (gal) (100)	Blueberry	Anti-inflammatory, anti-angiogenesis	**T1**: 1, 10, 50 + 100 µM glc, **T2**: 1, 10, 50 + 100 µM gal, **T3**: mixture of T1-T2, **U**: TNF stressed, **C**: DMSO in unstressed cell.	HUVEC. **S**: 10 µg/L TNF-α for 6h.	The best was T1 in a concentration-dependent manner. T1 + T2 ↓genes and proteins: TNF-α, MCP-1, ICAM-1, VCAM-1, IκBα. T1 + T2 inhibits nuclear translocation of p65.	[41]
Chalcone	Isoliquiritigenin (> 98)	Liquorice root	Anti-angiogenesis, anti-apoptotic, anti-inflammatory	**T**: 0, 2, 10, 25, + 50 µM, **C**: normal saline with 0.01% DMSO in stressed cell.	HUVEC. **S**: 312 µg/mL recombinant VEGF for 24h.	T is beneficial. ↓vascular leakage. ↓neovascular area in all samples. ↓VEGF + ↑PEDF expression.	[42]
Flavan-3-ol	Epigallocatechin-3-gallate (> 98)	Not disclosed	Anti-inflammatory, anti-oxidation, anti-angiogenesis	**T**: 10, 20 + 30 µM, **U**: glucose stressed, **C**: DMSO in unstressed cell.	Mouse primary retinal Müller cells. **S1**: 30 mM glucose. **S2**: 200 µM TBHP + 5 mM ATP for 24h.	T is beneficial. ↓ROS/TXNIP/NLRP3 inflammasome axis. ↓MDA + ↑ SOD. ↓VEGF and HGF.	[43]
Flavone	Chrysin	Not disclosed	Anti-angiogenesis, anti-inflammatory	**T**: 1, 10 + 20 µM, **U**: glucose stressed, **C**: 5 mM glucose + 27.5 mM mannitol in healthy cell.	HRMVEC. **S**: 33 mM glucose for 72h.	T is beneficial. ↓endothelial apoptosis in the retina induced by excess glucose. ↓HIF-1α + VEGF. ↓Ang-1, Ang-2, + Tie-2 level. ↓N-cadherin + ↑endothelial VE-cadherin.	[44]
	Nepetin	Japanese elecampane	Anti-inflammatory	**T**: 2.5, 5 + 10 µM, **U**: IL-1β stressed, **C**: DMSO in unstressed cell.	ARPE-19. **S**: 10 ng/mL IL-1β for 0.5h.	T is beneficial. ↓IL-6, IL-8, + MCP-1 at protein and mRNA level. ↓nuclear translocation of p65 with suppressive phosphorylation of IκB + IKK. ↓phosphorylation of ERK 1/2, JNK + p38 MAPK	[45]
Flavonols	Dihydromyricetin (> 98)	Not disclosed	Anti-apoptotic, anti-oxidation	**T**: 30, 100 + 300 µmol/L, **U**: glucose stressed, **C**: 5.5 mmol/L glucose + 22.5 mmol/L mannitol in unstressed cell.	ARPE-19. **S**: 25 mmol/L glucose for 48h.	T is beneficial. ↑SOD, CAT, + GSH. ↓Bax, caspase-3, + cleaved caspase-9. ↑Bcl2 compared to the control. ↓miR-34a expression compared to control	[46]
	Galangin (> 98)	Not disclosed	Anti-apoptotic, anti-oxidation anti-inflammatory	**T**: 20 + 50 µM, **U**: glucose stressed + TNF stressed, **C**: 25 mM mannitol in unstressed cell.	HREC + ARPE-19 + Microglia BV2 cells. **S**: 25 mM glucose + 20 ng/ml TNFα for 24h.	T is beneficial. ↓cellular ROS formation in BV2. ↓phosphorylation of cRaf + MEK1/2, ERK1/2 in BV2 cell. ↓permeability of glucose stressed BV2 cell + HRECs. ↓permeability of glucose stressed BV2 + ARPE-19. ↑occludin + claudin1 in TNF-induced ARPE-19. ↓microglia activation, + ↓ ROS in BV2 cell. ↑accumulation of Nrf2 in nucleus. ↑nuclear phosphorylated Nrf2. ↓TNFα, IL-1β + IL-6 (mRNA) in BV2 cell. ↓p-IKK, p-IκB + p-NFκBp65 expression in BV2 cell. ↓nuclear accumulation of NFκBp65 + Egr1 in TNF induced HREC, ARPE-19.	[47]
	Icariin (> 98)	Not disclosed	Neuroprotective, anti-angiogenesis	**T**: 0, 10, 100 + 1000 nmol/mL, **U**: glucose stressed, **C**: normal saline + DMSO in unstressed cell.	RGC. **S**: High glucose for 3d.	T is beneficial. ↓VEGF, Brn3a, Thy-1, + RECA (protein). ↓collagen IV + Müller cell content. ↑neurite outgrowth compared to control.	[48]
	Kaempferol (> 98)	Not disclosed	Anti-angiogenesis, anti-proliferative	**T**: 5–25 µM, **U**: glucose stressed, **C**: 5 mM normal glucose in unstressed cell.	HREC. **S**: 25 mM glucose for 12–48h	T is beneficial. ↓cell proliferation, migration, migration distance, + sprouting. ↓PI3K, Erk1/2 activation, Src, + Akt1. ↓VEGF + PGF (mRNA).	[49]
Homoisoflavonoid	Cremastranone (> 98)	Not disclosed	Anti-angiogenesis, anti-apoptotic, anti-proliferative, anti-inflammatory	**T1**: 0.5 µM SH-11,037, **T2**: 50, 200, 400 + 800 µg/mL of aflibercept, **T3**: combination of SH-11,037 + aflibercept, **U**: VEGF stressed, **C**: DMSO in VEGF stressed cell.	HREC. **S**: 50 ng/mL recombinant VEGF for 24h	T1-T3 is beneficial. T1-T3 ↓CNV, hyaloid vessel, + branch development. No significant increase in GFAP, cleaved caspase 3, TUNEL, + MCP1 in T1-T3. Absence of toxicity in T1. ↓HREC proliferation for T3 > each dose of T2. Synergistic effects of T3 ≥ 0 for excess % inhibition over the highest single agent (HSA) + excess over bliss additivity.	[50]
	Homoisoflavonone (> 98)	Chinese cremastra	Anti-angiogenesis, anti-proliferative	**T**: 1–10 µM, **U**: FGF-2 stressed, **C** = 1 µL PBS in stressed cell.	HUVEC. **S**: 1 µM FGF-2 for 18h.	T is beneficial. ↑neovascular lumens. ↓CNV + vascular leakage. ↓tube formation, + capillary-like network. Normal retina thickness. ↓FGF-2-induced HUVECs migration. No effect on cell viability. Retinal toxicity absent	[51]
Isoflavonoid	Deguelin (> 98)	Not disclosed	Anti-angiogenesis, anti-proliferative	**T**: 0, 0.01, 0.1, 1 + 10 µM. **U**: hypoxia stressed. **C**: 0.1% DMSO in unstressed cell.	HREC. **S**: 5% CO_2_, 1% O_2_ + N2-induced for 24h.	T is beneficial. ↓VEGF + HIF-1α. ↓clinically significant vascular leakage. No effect on cell viability.	[52]
	Formononetin (> 98)	Mongolian milkvetch	Anti-angiogenesis, anti-apoptotic, anti-inflammatory	**T1**: 0.2, 1 + 5 µg/mL, **T2**: 1 µg/Ml YC-1, **C**: DMSO in unstressed cell.	ARPE-19. **S**: 150 µM CoCl_2_ for 24h.	T1 + T2 is beneficial. T1 + T2 ↓VEGF, VEGFA, HIF-1α (protein), + PHD-2 (mRNA) level. T1 + T2 ↓NV + reverse of the hypoxia-induced neovascular area ratio	[53]
	Genistein (> 98)	Not disclosed	Anti-inflammatory, anti-angiogenesis	**T1**: 0, 10, 20, 50 + 100 µM, **T2**: 10 µM U0126, **T3**: 10 µM SB203580, **U**: glycated albumin stressed, **C**: Non-glycated albumin in unstressed cell.	Primary retinal microglia culture. **S**: 10–500 µg/mL glycated albumin for 24h	T1-T3 is beneficial. T1-T3 ↓ERK phosphorylation. T1 ↓TNF-α in a dose-dependent manner. T1 ↓ERK + P38 MAPKs compared to T2, T3 + control. No effect on cell viability in T1 (88–94% vital cells).	[54]
Hydroxycinnamic acid	Curcumin (> 98)	Ginger	Anti-diabetic, anti-angiogenesis, anti-oxidation, anti-apoptotic	**T**: 1, 3, 10 + 30 µM, **U**: glucose stressed, **C**: no stressor.	HREC. **S**: 30 mM glucose for 72h.	T is beneficial. ↓blood glucose level ↓VEGF (65%) + PKC (31%) expression. ↓intracellular ROS (LDH). ↑caspase-3.	[55]
Stilbene	Resveratrol (> 98)	Red wine and grape	Anti-angiogenesis, anti-oxidation, anti-apoptotic	**T**: 1, 5, 10 + 20 µM, **U**: glucose stressed, **C**: 5 mM glucose + 25 mM mannitol in unstressed cell.	BREC. **S**1: 30 mM glucose. **S2**: 5 mM H_2_O_2_ for 48h	T is beneficial. ↑p-AMPK ↓Sirt1 + PGC-1α ↓ROS + caspase. No effect on cell viability	[56]

Abbreviations: AA: ascorbic acid, TBHP; tert-butyl hydroperoxide, AAPH: 2,2’-azobis(2amidinopropane) dihydrochloride, AKT: serine/threonine kinase, ALR: aldose reductase, AMPK: adenosine monophosphate-activated protein kinase, Ang1/2: Angiopoietin 1/2, ATP: adenosine triphosphate, Bax: bcl-2 associated x-protein, Bcl2: b-cell lymphoma protein 2, CAT: catalase, CNV: choroidal neovascularisation, CoCl_2_: cobalt(II) chloride, DMSO: dimethyl sulfoxide, eNOS: endothelial nitric oxide synthase, ERK1/2: extracellular signal-regulated protein kinase1/2, ET-1: endothelin-1, FGF-2: fibroblast growth factor, FGF-2: fibroblast growth factor, G6PDH: glucose-6-phosphate dehydrogenase, GFAP: glial fibrillary acidic protein, GFAP: glial fibrillary acidic protein, GSH: glutathione, H2O2: hydrogen peroxide, HIF-1α: hypoxia-inducible factor 1 alpha, HMGB1: high mobility group box 1 protein, ICAM-1: intercellular adhesion molecule-1, IL-1β: interleukins 1 beta, iNOS: inducible nitric oxide synthase, JNK: c-Jun n-terminal kinase, MCP1: monocyte chemotactic protein-1, MDA: malondialdehyde, MMP-9: matrix metalloproteinase-9, NF-κB: nuclear factor-kappa B, NT: 3-nitrotyrosine, NV: neovascularisation, p38MAPK: p38 mitogen-activated protein kinases, p70 S6K: protein 70 S6 kinase, p-AMPK: phosphorylated amp-activated protein kinase, PEDF: pigment epithelium-derived factor, PGC-1α: peroxisome proliferator-activated receptor-γ coactivator 1α, PHD-2: prolyl hydroxylase domain-containing protein 2, PI3K: phosphatidylinositol-3-kinase, PKC/β2: protein kinase C/beta 2, RAGE: receptor for advance glycation endproducts, ROS: reactive oxidative species, S100B: calcium binding protein B, SB20358: P38 inhibitor, SDH: sorbitol dehydrogenase, Sirt1: sirtuin 1, SOD: superoxide dismutase, STAT3: signal transducer and activator of transcription 3, TGF-β1/β2: transforming growth factor-beta 1/beta 2, Tie-2: tyrosine kinase with immunoglobulin-like and egf-like domains, TNF-α: tumor necrosis factor alpha, TUNEL: terminal deoxynucleotidyl transferase (tdt) dutp nick-end labeling, U0126: erk inhibitor, VEGF/R2: vascular endothelial growth factor/receptor 2, YC-1: 5-[1(phenylmethyl)-1 H-indazol-3-yl]-2-furanmethanol

↑: increase/upregulate, ↓: decrease/downregulate, m; month, d; day, h; hour, w; week, Dose* = T; treated, C: control, S: stressor. Cell culture models were associated with for microvascular disease due to responsiveness to retinopathy. Cell abbreviations: BV2: mouse microglial cell line, BAEC: bovine aortic endothelial cells, BREC: bovine retinal endothelial cells, HAEC: human aortic endothelial cells, HUVEC: human umbilical vein endothelial cell, HREC: human retinal endothelial cells, ARPE-19: retinal pigment epithelial cell line, HRMVEC: primary human retinal microvascular endothelial cells

**Table 3:** In-vivo studies of polyphenols for the treatment of early retinopathy

Polyphenol	Subclass of compound (%purity)	Natural Source	Target action classification	Dose*	Retinopathy model/ stressor	Outcome of polyphenol treatment	References
Anthocyanin	Anthocyanin-rich bilberry extract (39)	Bilberry	Anti-inflammatory, anti-oxidation	**T**: 500 mg/kg. **U**: DR stressed, **C**: PBS in normal mice for 4d	C57BL/6 mice (6w) sex unspecified. **S** = 6 mg/kg LPS by ip at 3h	↓STAT3 activation *+* IL-6 + NF-*κ*B p56. Reduces intracellular ROS	[57]
	Epicatechin (EC), epigallocatechin (EGC), epicatechin gallate (ECG), and epigallocatechin-3-gallate (EGCG)	Green tea	Anti-inflammatory, anti-oxidation	**T**: 550 + 275 mg/mL GTE. **U**: DR stressed + fed with water, **C**: saline injection in normal rat + fed with water at 2, 8, 26 + 36h	Male Sprague-Dawley adult rat (age not stated). **S** = 1 mg/kg LPS by if	↓IL-1β, TNF-α, + IL-6 in the retina + vitreous humour. ↓phosphorylation of STAT3 + NF-κB in the retina + receptor-mediated action on transcription factors	[58]
Flavanone	Eriodictyol (> 98)	Not disclosed	Anti-inflammatory, anti-oxidation	**T**: 0.1, 1, 10 mg/kg ERI. **U**: DR stressed, **C**: citrate buffer in normal rat, orally daily for 10d	Male Sprague-Dawley rat (age not stated). **S** = 60 mg/kg STZ by iv at 12h	Effective = 10 mg/kg ↓retinal TNF-α, ICAM-1, VEGF, + eNOS in a dose-dependent manner in the diabetic control. ↓lipid peroxidation + BRB breakdown.	[59]
	Hesperidin (Hsp) (> 98)	Not disclosed	Anti-angiogenesis, anti-inflammatory, anti-oxidation	**T**: 100 + 200 mg/kg. **U**: DR stressed, **C**: calcium dobesilate injected in normal + DR stressed rat. 1ce for 12w ig	Male Sprague-Dawley rat (age not stated). **S** = 60 mg/kg STZ by ip at 12h	↓BRB breakdown (35%:Hsp 100 mg/kg, 48%:Hsp 200 mg/kg and 42%: CaD) + ↑ retina thickness. ↓blood glucose, retinal VEGF, TNF-α, ICAM-1, IL-1β, + AGEs levels. ↓plasma MDA + ↑ SOD. ↓ALR compared to untreated.	[60]
	Hesperetin (> 98)	Not disclosed	Anti-inflammatory, anti-oxidation, anti-autophagy	**T**: 50 + 100 mg/kg. **U**: DR stressed, **C**: 1 mL/kg of citrate buffer in normal rat. Orally/d for 6w	Male Wistar albino rats (age not stated). **S** = 30 mg/kg STZ injection twice after 8d	Effective = 100 mg/kg. ↓retinal TNF-α, IL-1β, IL-6, + NFκB expression (mRNA + protein). ↓ p62. ↑defective beclin 1 + LC3-II. ↓serum AGE + retinal MDA. ↓area percentage of PAS-positive reaction for 100 mg/kg like vehicle group. ↓RCL, ONL, + INL thickness compared to the vehicle group. normal structure of retina.	[61]
	Naringenin (> 98)	Not disclosed	Anti-diabetic, anti-oxidation, anti-apoptotic	**T**: 50 mg/kg/day. **U**: DR stressed, **C**: citrate buffer for normal + diabetic rat. orally for 5w	Male Wistar albino rats (12w). **S** = 65 mg/kg STZ by ip	↑BDNF in treated by 7 pg/µg compared to untreated diabetic with 5 pg/µg. ↑TrkB, Bcl-2. ↓Bax, + caspase-3 compared to the control ↑retinal GHS compared to the untreated diabetic ↓TBARS compared to the untreated diabetic	[62]
Flavan-3-ol	Catechin (> 98)	Green tea	Anti-inflammatory	**T**: 50, 100 + 200 mg/kg/day. **U**: DR stressed, **C**: 5 mL physiological saline in normal rat. iv for 8w	Male Sprague-Dawley rat (8w). **S** = 60 mg/kg STZ by ip	Effective = All T. ↑retinal HSP27 levels in a concentration-dependent manner (mRNA). ↓IL-1β, IL-6, + TNF-α compared to diabetic control. ↓NF-κB p65 + p-NF-κB p65 activation compared to the diabetic control	[63]
	Epigallocatechin-3-gallate (> 98)	Not disclosed	Anti-inflammatory, anti-oxidation, anti-angiogenesis	**T**: 1 g/kg/day pro-EGCG, + 1 mL/kg/day 0.1% DMSO. **U**: DR stressed, **C**: normal mice (standard diet) + 0.1% DMSO injected in diabetic mice fed with 42% high fat diet. Gavage for 1w	Male C57BL/6 mice (8w). **S** = 60 mg/kg STZ by ip	↓ROS. ↓TXNIP, NLRP3, ASC, cleaved caspase-1, pro-caspase-1, cleaved IL-1β, + pro-IL-1β protein. ↓VEGF + HGF production. ↓ROS/TXNIP/NLRP3 inflammasome axis activity	[43]
Flavones	Baicalein	Chinese skullcap	Anti-inflammatory, anti-angiogenesis	**T**: 75, 150, + 300 mg/kg/d. **U**: DR stressed, **C**: sodium citrate buffer in normal rat. Orally/d for 24w	Female rat (age not stated). **S** = 60 mg/kg STZ by ip at 16h	Effective = 150 mg/kg. ↑GCL cell (48%) than diabetic untreated (27 vs. 18). ↓microglial activation. ↓GFAP + VEGF compared to the untreated diabetic. ↓IL-18, TNF-α, + IL-1β compared to untreated diabetic retina. ↓vascular changes.	[64]
	Chrysin	Not disclosed	Anti-angiogenesis, anti-inflammatory	**T**: 10 mg/kg. **U**: DR stressed, **C**: normal mice. Orally daily for 10w	Male db/db + db/m adult mice (7w). **S** = genetic	↑PECAM-1 + endothelial VE-cadherin. ↓HIF-1α + VEGF and VEGFR2. Restored ZO-1 protein. Fewer neovascular tufts + lack of diffuse retina. ↓vascular permeability	[44]
	Luteolin (> 98)	Not disclosed	Anti-oxidation, anti-apoptotic	**T**: 50 mg/kg. **U**: DR stressed, **C**: normal rat. Orally for 4w	Male Wistar rat (age not stated). **S** = 60 mg/kg STZ by ip	↓glucose concentration, retinal vessels diameter (46.1 µM), ↓MDA, + ↑ SOD compared to the untreated DR. ↓IL-1β, IL-6, TNF-α, +NF-kB. ↓Bax, + caspase-1, + ↑ Bcl-2. ↓NLRP1, NOX4, TXNIP, + NLRP3 compared to the untreated DR	[65]
	Scutellarin	Barbated skullcap	Anti-inflammatory, anti-angiogenesis	**T**: 100 + 200 mg/kg. **U**: DR stressed, **C**: physiological saline injected in normal mice. Orally for 1m	Male C57BL/6J mice (age not stated). **S** = 55 mg/kg STZ by ip daily for 5d	Effective = 200 mg/kg. ↓TNF-α + IL-1β retinal, mRNA. ↓ICAM-1, + phosphorylation of NFκB p65. ↑claudin-1 + claudin-19	[66]
	Silymarin (> 98)	Not disclosed	Anti-oxidation, anti-inflammatory	**T**: 50 + 100 mg/kg. **U**: DR stressed + distilled water, **C**: distilled water in normal rat. Orally 1ce daily for 8w	Male Wistar rat (2 m). **S** = 60 mg/kg STZ by iv	Effective = All T ↓AGEs + RAGE. ↓phosphorylation of p38MAPK + NF-κB p65. ↓ROS. ↓IL-1β, IL-6 + TNF-α (transcript). ↓VEGF, ECM (protein). ↓VCAM-1 + ICAM-1 (mRNA). ↓TGF-β, collagen IV, + fibronectin compared to untreated diabetic (mRNA)	[67]
Flavonols	Galangin (> 98)	Lesser galangal	Anti-apoptotic, anti-oxidation, anti-inflammatory	**T**: 1, 10 mg/kg. **U**: DR stressed, **C**: physiological saline injected in normal mice. Orally for 1m	Male C57BL/6 mice (age not stated). **S** = 55 mg/kg STZ by ip daily for 5d	Effective = 10 mg/kg. ↓microglia cell activation, ↓ROS, ERK 1/2 phosphorylation, + NFκB. ↓TNFα, IL-1β, IL-6 (mRNA), + Egr1 protein. ↑claudin1 + occludin ↓BRB breakdown. ↓Iba1 protein expression in retina + microglia cells in OPL, IPL + GCL. ↓phosphorylation of cRaf, MEK1/2 + ERK1/2 in retinas. ↓evans blue dye leakage from retinas in wild-type mice, + no reduction from retinas in Nrf2 knockout mice.	[47]
	Icariin (> 98)	Horny goat weed	Anti-apoptotic, neuroprotective	**T**: 5 mg/kg/day. **C**: physiological saline + DMSO injection in diabetic rat (placebo). Gavage for 12w	Male Sprague-Dawley rat (8w). **S** = 60 mg/kg STZ by ip	↑thickness of retina basal membrane. ↑VEGF, + ↓ RECA compared to the placebo (protein). ↓Collagen IV + Müller cell content. ↑neurite outgrowth from treatment. ↑Thy-1 + Brn3a in the INL, ONL, RGCs compared to placebo retinas.	[48]
	Quercetin (> 98)	Not disclosed	Anti-oxidation, anti-angiogenesis, neuroprotective	**T1**: 150 mg/kg, **T2**: 150 mg/kg + 30 mg/kg Znpp **U**: DR stressed, **C**: physiological saline injected in normal rat. Note: Quercetin 1ce daily ig + Znpp 1ce at 10 am every 2w for 16w	Male Sprague-Dawley rat (8w). **S** = 60 mg/kg STZ by ip	Effective = T1 T2 Znpp blocks benefit of quercetin. ↑thickness of the retinal cell layer, + number of GCLs. ↓IL-1β, IL-18, IL-6, + TNF-α in the retinal. ↑HMGB1 + NLRP3 inflammasome mRNA and protein in Znpp treated group than only quercetin. ↓TLR4 + NF-κBp65. ↓VEGF + ICAM-1 expression. ↑BDNF (dependent on HO-1) + NGF.	[68]
	Rutin (> 98)	Not disclosed	Neuroprotective, anti-apoptotic, anti-oxidation, anti-diabetic	**T**: 100 mg/kg. **U**: DR stressed + saline, **C**: physiological saline injected in normal rat. Orally for 5w	Male Wistar rat (age not stated). **S** = 65 mg/kg STZ by ip	↓glucose level. ↑insulin production. ↑BDNF + GSH. ↓TBARS + caspase-3. ↑Bcl-2 compared to the untreated diabetic rats.	[69]
Isoflavonoid	Biochanin A (> 98)	Red clover, cabbage, and alfalfa	Anti-angiogenesis, anti-inflammatory, anti-diabetic	**T**: 10 + 15 mg/kg. **U**: DR stressed, **C**: 0.5% DMSO injected in normal rat. Orally for 6w	Male Wistar rat (age not stated). **S** = 55 mg/kg STZ by ip	Effective = 15 mg/kg. ↓glucose level. ↓VEGF, TNF-*α*, + IL-1β. Reverse BRB breakdown compared to diabetes vehicle-treated	[70]
	Daidzein (> 98)	Kwao krua and kudzu root	Anti-oxidation, anti-angiogenesis, anti-diabetic	**T**: 25, 50 + 100 mg/kg. **U**: DR stressed, **C**: 0.5% sodium carboxymethyl cellulose injected in normal rat for 28d	Male Sprague-Dawley rat (age not stated). **S** = 55 mg/kg STZ by ip	Effective = 100 mg/kg. ↓electric response of α-wave amplitude (− 59 µV) + β-wave amplitude (206 µV) in the retina compared with normal control of α-wave (− 148 µV) and β-wave (317 µV). ↓elevated blood glucose. ↓ALR + SDH, + MDA. ↑GSH, SOD + CAT. ↓retinal thickness compared to diabetes control	[71]
	Deguelin (> 98)	Not disclosed	Anti-angiogenesis, anti-proliferation	**T**: 0.1 µM + 1 µL. **U**: Hypoxia stressed, **C**: PBS injected in normal mice. iv for 4d	Female C57BL/6 mice (age not stated). **S** = 75% of O_2_ for 5d	Effective = All T. ↓intravitreous NV. ↓vascular leakage compared to the control	[52]
	Formononetin (> 98)	Mongolian milkvetch	Anti-angiogenesis, anti-apoptotic	**T**: 1, 5 + 10 mg/kg. **U**: Hypoxia stressed, **C**: physiological saline injected in norma rat. ip for 5d	Pregnant female Sprague-Dawley rat (16-20w) + pups. **S** = 80% of O_2_ for 5d	Effective = All T. ↓NV area ratio effect on the retina. ↓hypoxia-induced neovascular area ratio. ↓vessel tortuosity, + dilated vessels. ↓neovascular nuclei + vessels in the retinal	[53]
	Genistein (> 98)	Not disclosed	Anti-inflammatory, anti-angiogenesis	**T**: 50 µM. **U**: DR stressed, **C**: 0.05% DMSO injected in normal rat. iv 1ce for 8h	Male Sprague-Dawley rat (age not stated). **S** = 60 mg/kg STZ iv for 2w	↓TNF-α (protein and mRNA). ↓inflammation by dampening microglial cell activation. ↓Iba1 (75% mRNA) compared to the vehicle treated. ↓ERK + P38 MAPKs compared to the vehicle treated.	[54]
	Puerarin (> 98)	Kudzu root	Anti-angiogenesis, anti-inflammatory	**T**: 80 mg/kg. **U**: DR stressed, **C**: physiological saline injected in normal rat. ip for 1, 3 + 5m	Male Wistar rat (age not stated). **S** = 60 mg/kg STZ by ip	↓morphological changes in the retina’s INL + ONL compared to the untreated. ↓VEGF + HIF-1α (mRNA) compared to the untreated (in 1–5 m)	[72]
Hydroxycinnamic acids	Curcumin (> 98)	Turmeric	Anti-angiogenesis, anti-oxidation, anti-apoptotic, anti-diabetic	**T**: 100 + 200 mg/kg. **U**: DR stressed, **C**: carboxymethyl cellulose sodium injected in normal rat. Orally for 16w	Male wistar rat (age not stated). S = 40 mg/kg STZ by ip	Effective = 200 mg/kg. ↓blood glucose levels. ↓thickening of the retinal capillary basement membrane. Orderly photoreceptor cell ↑thickness of the overall retina (44.75 ± 0.35 μm) compared to diabetic (42.13 ± 0.49 μm). ↓MDA + ↑ SOD + T-AOC. ↓VEGF, ↑ Bcl-2. ↓Bax expression.	[73]
Stilbene	Resveratrol (> 98)	Not disclosed	Anti-angiogenesis, anti-oxidation, anti-apoptotic	**T**: 5 mg/kg. **U**: DR stressed, **C**: citrate buffer injected in normal rat normal rat. Orally for 4m	Male wistar rat (age not stated). **S** = 50 mg/kg STZ by ip at 12h	↓blood glucose + weight loss in diabetes treated compared to diabetes control. ↑SOD blood and retinas compared to diabetes control. ↓NF-κB + apoptosis rate compared to diabetes control in retina. ↓retinal layer disarrangement. ↑retinal thickness compared to diabetes control	[74]

Abbreviations: AGE: advance glycation endproducts, ALR: aldose reductase, AUC: area under the curve of plasma concentration as a function of time, Bax: bcl-2 Associated X-protein, Bcl2: b-cell lymphoma protein 2, BDNF: brain-derived neurotrophic factor, BRB: blood retinal barrier, Brn3a: brain-specific homeobox/pou domain protein 3a, CAT: catalase, CD68: a cluster of differentiation 68, Cl: clearance, C_max_: maximum plasma concentration, c-Raf: proto-oncogene serine/threonine protein kinase, CS-SA LNC: chitosan-sodium alginate lutein-loaded nanocarrier system, DMSO: dimethyl sulfoxide, ECM: endothelial cell membrane, egr1: early growth response 1, ERK: extracellular signal-regulated kinase, EYA3: expression of eyes absent, FGF-2: fibroblast growth factor, FKN: fractalkine, GCL: ganglion cell layer, GFAP: glial fibrillary acidic protein, GSH: glutathione, GTE: green tea extract, HAS: highest single agent, HO-1; heme oxygenase-1, HSP27: heat shock protein 27, Iba-1: ionized calcium-binding adapter molecule 1, ICAM-1: intercellular adhesion molecule-1, IL-1β: interleukins 1 beta, INL: inner nuclear layer cells, iNOS: inducible nitric oxide synthase, IPL: inner plexiform layer, LDH: lactate dehydrogenase, LPS: lipopolysaccharide, MCP-1: monocyte chemoattractant protein-1, MCP1: monocyte chemotactic protein-1, MDA: malondialdehyde, MRT: mean resident time, Mv-gal: malvidin-3-galactoside, Mv-glc: malvidin-3-glucoside, NF-κB: nuclear factor-kappa B, NGF: nerve growth factor, NV: neovascularisation, OPL: outer plexiform layer, p38 MAPK: p38 mitogen-activated protein kinases, PBS: phosphate buffered saline, PEDF: pigment epithelium-derived factor, PL: phospholipid nano-carrier, PRDX: peroxiredoxin, RAGE: receptor for advance glycation endproducts, RECA: rat endothelial cell antigen protein, ROS: reactive oxidative species, SDH: sorbitol dehydrogenase, sICAM-1: soluble intercellular adhesion molecule-1, SOD: superoxide dismutase, STAT3: Signal transducer and activator of transcription 3, STZ: streptozotocin, T_1/2_: half-life, T-AOC: total antioxidant capacity, TBARS: thiobarbituric acid reactive substances, TEER: transendothelial electrical resistance, Thy1: thymocyte antigen 1, TNF-α: tumour necrosis factors alpha, VEGF: vascular endothelial growth factor, VCAM-1: vascular cell adhesion molecule-1

↑: increase/upregulate, ↓: decrease/downregulate, m; month, d; day, h; hour, 1ce: once. Dose* = T; treatment, U: untreated, C: control, S: stressor, ip: intraperitoneal, iv: intravitreal, ig: intragastric, if: injection in footpad

### Carotenoids

**Fig. 2:**
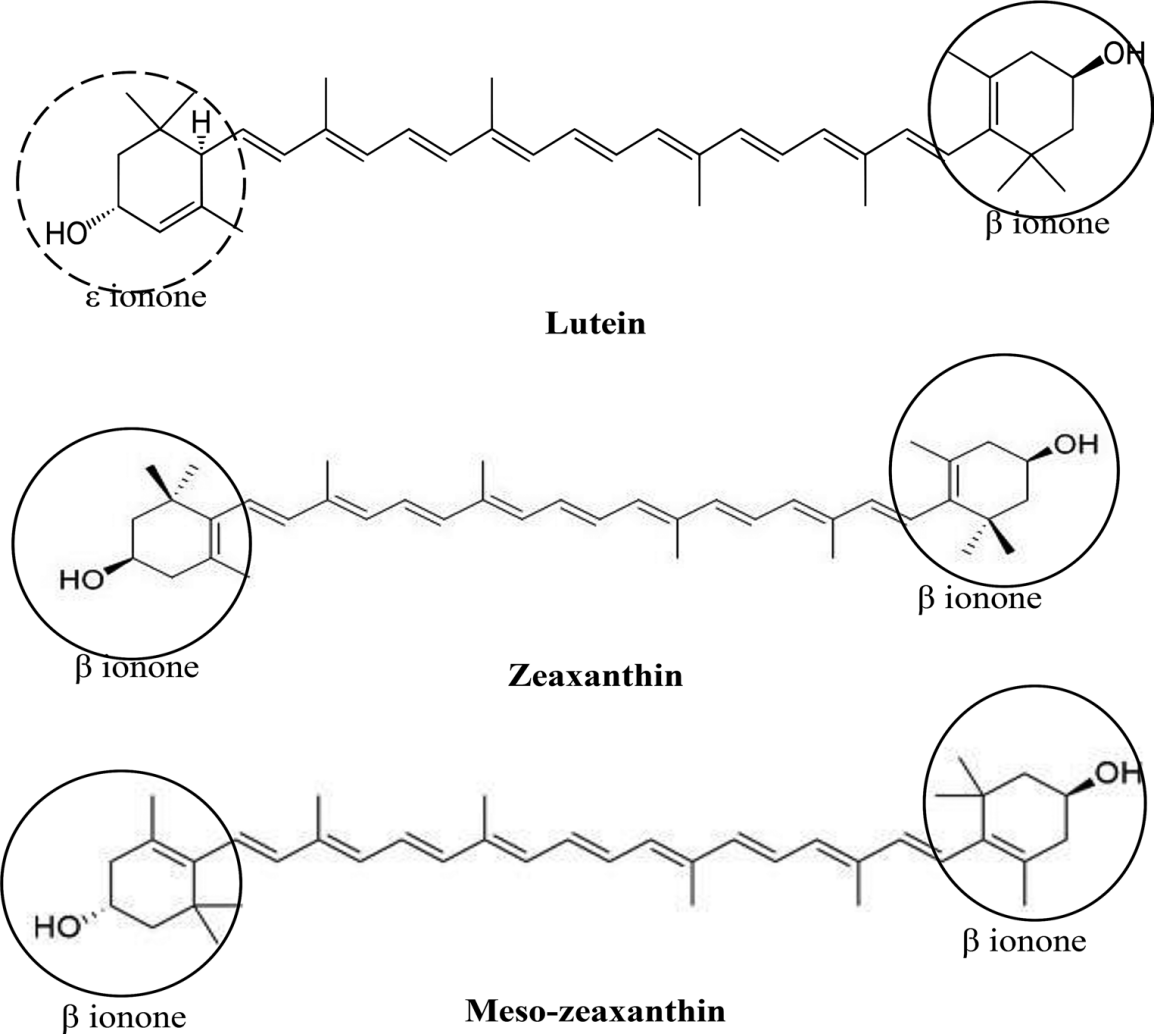
The chemical structure of important macular pigments: lutein, zeaxanthin and meso-zeaxanthin that confer protection to the retina against retinopathy damage

Carotenoids are naturally occurring isoprenoid pigments found in various plants and microalgae. There are over 750 naturally occurring carotenoids structurally classified under carotene (non-oxygen containing) and xanthophyll (oxygen containing) in plants. Among the xanthophylls, only lutein and zeaxanthin bioaccumulate in the central retina area (macula) as yellow colouration visible with ophthalmoscopy and are termed macular pigments (MP) [75]. The MP are therefore prioritised for maintaining eye health over other bioactive compounds. The lutein and zeaxanthin are 40-carbon chains with nine conjugated double bonds in the polyene chain. As isomers (not stereoisomers), they are structurally different by the presence of a β-ionone ring and an ε-ionone ring in lutein but two β-ionone rings for the zeaxanthin (Fig. 2), and this implicates their antioxidation and light filtering capabilities [76]. Importantly, these carotenoids can filter blue and UV light from artificial electronic sources or natural direct sunlight exposure capable of inducing photodamage, oxidative stress and inflammation which lead to solar/radiation retinopathy [77, 78]. With the rapid increase of electronic device usage that exerts additional oxidative and inflammatory stress on retinal tissues, over time this could exacerbate retinopathy pathology [79], that could be alleviated with macular pigments as natural filters of high-energy blue light [77]. These multiple benefits of carotenoids and its multiple pathways of action underscore their significant role in preventing and mitigating chronic eye diseases.

Another member of MP is meso-zeaxanthin, which is not readily available from natural sources but is biosynthesised from isomerisation of lutein in the macular. Aside from meso-zeaxanthin, the other two MP are highly valued in protecting the eye, and the percentage of lutein and zeaxanthin in the serum accounts for 20–30% of the total carotenoids and is even higher in the retina accounting for 80–90%. More so, the ratio of both is about the same (1:1) in the macular but lutein predominates zeaxanthin (3:1) in the peripheral retinal across all age groups [75].

**Fig. 3:**
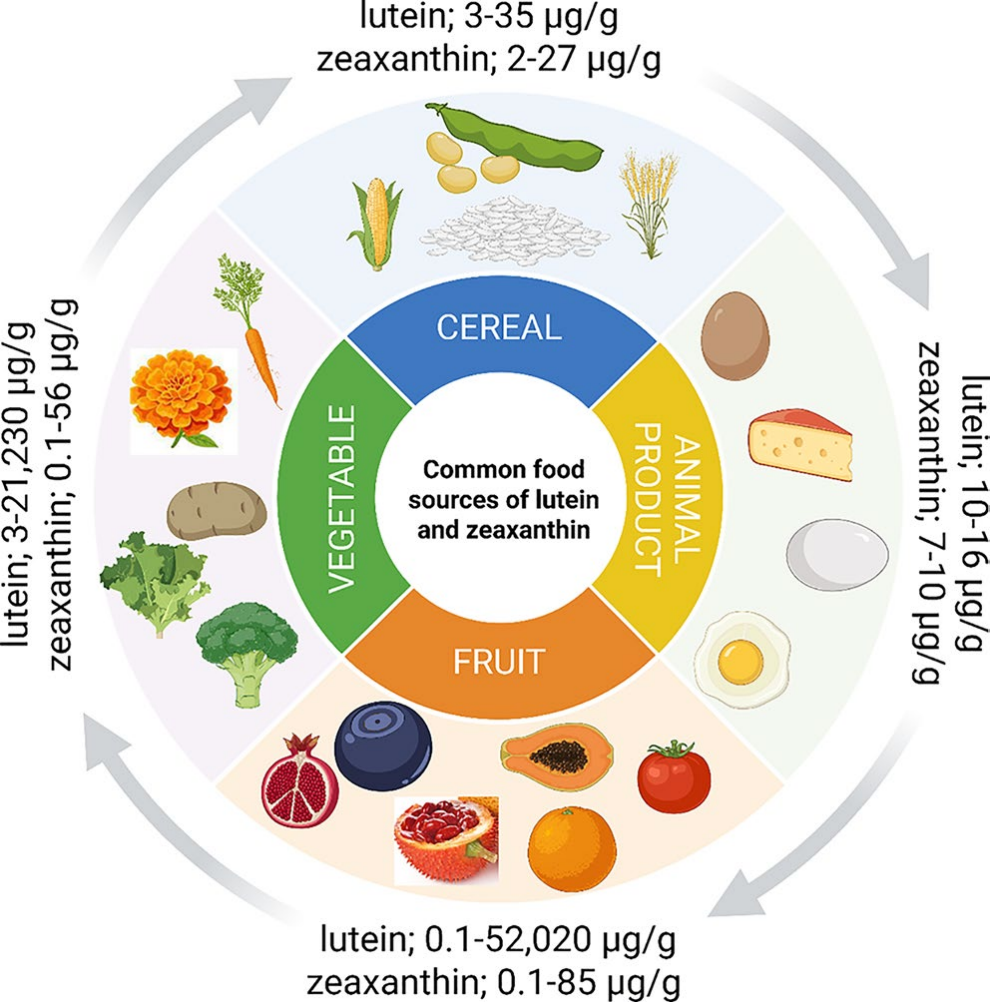
Common food sources of lutein and zeaxanthin from vegetables, fruits, cereal, and animal products. Created in Biorender

Lutein and zeaxanthin are found in commonly eaten green leafy vegetables, fruit, cereal, and animal products like eggs (Fig. 3) [80]. At present, lutein and zeaxanthin is well recognised for eye health [81, 82] and categorised as safe (GRAS) as dietary supplements since 2001 [83], however their general benefit on human health also transcends to cancers, stroke, obesity, and coronary heart diseases [84, 85]. This makes it quite important to distinguish carotenoid-rich plant sources, where marigold flowers (*Tagetes erecta*) remain the gold standard for lutein and zeaxanthin extraction with lutein esters making up almost 90% (~ 21,230 µg/g) of the total carotenoids [86]. In some common dietary crops, the extraction of lutein and zeaxanthin yielded low (< 5 µg/g), moderate (5–20 µg/g) and high (> 20 µg/g) concentrations, and this is less compared to marigold [87]. To date, only gac fruit peels recorded 2.5 times higher lutein (52,020 µg/g) than marigold but it is often discarded as waste [88]. Hence, upcycling of gac fruit peel represent a viable source of lutein and other carotenoids to meet the standard recommended amount needed for daily intake [87].

Grains used as cereal like millet, sorghum, maize, wheat barley, and rice are natural sources of lutein and zeaxanthin [89]. The recovery quantification is low, due to a range of factors including preparation (raw, soaked, or boiled), plant developmental stage [90], and the extraction processes [91]. Carotenoid concentrations from cereal ranges 3–35 µg/g for lutein and 2–27 µg/g for zeaxanthin, and is low compared to fruits with 0.1–52,020 µg/g for lutein, 0.1–85 µg/g zeaxanthin, and vegetables with 3–21,230 µg/g for lutein and 0.1–56 µg/g for zeaxanthin, respectively [89], and thus makes cereal a suboptimal source of adequate lutein and zeaxanthin. For eggs, the concentrations are also inferior, with ranges from 10 to 16 µg/g for lutein, 7–10 µg/g for zeaxanthin, and traces of meso-zeaxanthin of about 0.1 µg/g [92].

## The effects of macular carotenoids on early retinopathy

### Carotenoid concentrations as indicators of eye health

The MPs are concentrated within the macula and is an important indicator for its critical role in eye health. The concentration of MP (lutein, zeaxanthin, and meso-zeaxanthin) in healthy patients is highest in the peripheral segment of the retina, the fovea, and macular epicentre, respectively [93]. When MP concentrations deplete in the retina, the neuronal and vascular layers of the retina becomes prone to light damage, oxidative stress, and inflammation-induced retinopathy, and this highlights the clinical significance of measuring MP optical density (MPOD) for proactive early interventions to replenish its level in the retina, serum, and plasma [94]. Therefore, the baseline of MP daily intake is used to guide supplementation or dietary management of retinal health, that is set at 2–10 mg for humans [95].

The biochemistry of carotenoid absorption across the blood retinal barrier (BRB), blood stream and cell membrane are facilitated by their binding to a specific transporter scavenger receptor class B type 1 and type 2 (SRB1/2), clusters of differentiation 36 (CD36), glutathione S-transferase 1 (GSTP1), steroidogenic acute regulatory domain (StARD) and tubulin. These proteins are found across the retinal pigment epithelium (RPE) cells, müller cells, and photoreceptors for the distribution of carotenoids based on their binding specificity, selectivity, affinity [93, 96, 97], and interactions with lipid soluble long aliphatic chains on low and high density lipo-proteins before reaching the macula to stimulate their protective functional activity against retinal diseases [93].

### Associations of carotenoids with visual function

In early DR, the initial functional deficits occur as inner neural retinal layer thinning and GCL loss. These changes can be measured with visual function tests which are useful for predicting the degree of neural damage including visual acuity (VA), MPOD, contrast sensitivity (CS), and glare sensitivity (GS), and optical coherence tomography (OCT) [98, 99]. Clinical studies of lutein, meso-zeaxanthin, and zeaxanthin (LMZ) supplementation (L; 6–10 mg/d, Z;0.5-2 mg/d and M;10 mg/d) showed significant benefits in slowing the progression of mild NPDR measured by VA and CS [100–102]. However, there are limited clinical evaluations on LMZ for the treatment of advance proliferative DR (PDR).

Additionally, two clinical LMZ interventions [103, 104], confirmed that the daily consumption of carotenoids (lutein; 10 mg, zeaxanthin; 2 mg and meso-zeaxanthin; 10 mg) improved visual function, with or without vitamins for 6 months and 2 years, respectively. Carotenoid supplementation increased the amount of MPOD by 27% in treated patients, compared to the placebo with 2% decrease in MPOD; and diets were specifically modified to restrict extra carotenoid-rich or omega-3 fatty acid-rich food intake throughout the study, ensuring that any observed effects could be strongly attributed to the administration of carotenoid supplementation [103]. This was supported by proteomic biomarkers that confirmed the benefit of LMZ supplementation for the diabetic subjects, resulting in 60% decrease of high-sensitivity C reactive protein (hsCRP), 9% decrease of low-density lipoprotein cholesterol (LDL-C), 9% decrease of triglyceride and 7% increase of high-density lipoprotein cholesterol (HDL-C) compared to the placebo with 11% decrease of hsCRP, 3% decrease of HDL-C, 1% increase of LDL-C, and 2% increase of triglycerides [103]. Therefore, this mechanistic evidence at the functional and proteomic levels involved in the pathogenesis of retinopathy strongly support the benefits of LMZ supplementation, even though there was a requirement for consistent, daily supplementation over an extended period (2 years) to observe improvements in visual function.

Overall, LMZ supplementation appeared promising based on 357 patients human clinical trials (Table 4), and the adverse effects were minimal, with only a single report of headaches in 1 out of 34 LZ treated patients [105]. However, there were several limitations of these studies; the sample sizes were generally small ranging from 15 to 60 patients; some studies lacked untreated retinopathy control groups for comparison in 4 studies [103, 104, 106, 107], and the addition of other supplements like multivitamin and mineral supplements that may interfere with the effects of LMZ [103]. Although the improvements of visual function from LMZ is relevant for physiological benefits, this alone is insufficient to establish a cause-effect relationship between LMZ supplementation and disease modification.

Therefore, the mechanistic evidence of biomarkers at the genetic and proteomic levels involved in oxidative, inflammatory, apoptosis, and angiogenesis stresses associated with retinopathy before and after administration of LMZ can help confirm the cellular changes before physical and functional manifestation. This offers independent biological evidence supporting the efficacy of herbal interventions, not only validating the clinical improvements observed but also identifying potential targets for preventative strategies.

**Table 4:** Human trials using lutein (L), Zeaxanthin (Z) and meso-zeaxanthin (M) in oral supplementation for retinopathy treatment

Patient selection criteria	Supplement	Experiment cohort	Dosage + duration	Outcomes of treatment	References
Presence of microaneurysms, haemorrhages, or hard exudates	L	**T**: 15 DR patients, **C**: 16 DR patients for placebo. All within 40-85y	10 mg/d L for 9m	L is beneficial. ↓ LogMAR by L improves VA = 0.08, ↑ LogMAR by placebo worsen VA = 0.02 at 36w. CS of L baseline = 1.05, after T = 0.16 change at 3cyc/d. CS of placebo baseline = 1.08, after T = 0.02 change at 3cyc/d. GS of L baseline = 0.98, after T = 0.07 change at 3cyc/d. GS of placebo baseline = 1.02, after T= -0.01 change at 3cyc/d. L was significant compared to the placebo.	[106]
Macular edema and macular foveal thickness	LZ	**T**: 34 DR patients (44-75y), **U**: 33 DR control patients (42-76y), **C**: 60 healthy patients (43-70y)	6 mg/d L + 0.5 mg/d Z for 3m	LZ is beneficial. Before T in serum. L + Z baseline = 0.23 µg/mL, + 0.0456 µg/mL normal control, L + Z baseline = 0.07 µg/mL, + 0.014 µg/mL DR treated, L + Z baseline = 0.071 µg/mL, + 0.01 µg/mL DR control. After T in serum. L + Z baseline = 0.54 µg/mL, + 0.28 µg/mL in DR treated, L + Z baseline = 0.07 µg/mL, + 0.02 µg/mL in DR control, BCVA baseline = 0.38, after T= ↑ BCVA 0.45 at 3 m. ↑ CS compared to baseline after T in 3 m. ↓ foveal thickness in 83% DR treated patient compared to the DR control	[105]
Within mild or moderate NPDR	LZ	**T**: 36 DR patients for LZ, **C**: 36 Z only patients. All within 40-75y	6 mg/d L, + 0.5 mg/d Z for 4m	LZ is beneficial. VA of LZ baseline = 0.37, after T = 0.44 µg/mL. VA of Z baseline = 0.35, after T = 0.39 µg/mL. CS of LZ baseline = 0.39, after T = 0.69 µg/mL. CS of Z baseline = 0.69, after T = 0.75 µg/mL. GS of LZ baseline = 0.99, after T = 0.70 µg/mL. GS of Z baseline = 1.02, after T = 0.77 µg/mL. No significant difference in VA, CS + GS before + after T	[107]
BCVA ≥ 8/10 in each eye, normal colour vision test and no signs of DR	LMZ	**T**: 60 DR patients for LMZ. All within 40-60y	10 mg/d L, 2 mg/d Z + 10 mg/d M for 48m	LMZ is beneficial. ↑ OD = 157.4 ± 13.7 baseline, to 162.8 ± 13.1 after T. ↑ OS = 157 baseline to 163 after T. ↑ RRD in all 3 central rings + no adverse effect experience. Absence of intraretinal fluid or cystic changes in the retina	[104]
With NPDR, DME or With no DR	LZ + mvm	**T**: 30 DR patients, for LZ + mvm. **C**: 37 placebo patients	Dose not specified. Treated for 6m	LZ is beneficial. LZ + mvm= ↑ 27% MPOD, placebo= ↓ 2% MPOD LZ + mvm= ↓ 60% hsCRP, placebo= ↓11% hsCRP LZ + mvm= ↓ 90% LDL-C, placebo= ↑ 1% LDL-C LZ + mvm= ↑ 7% HDL-C, placebo= ↓ 3% HDL-C, LZ + mvm= ↓ 8.6% triglyceride, placebo= ↑ 2% triglyceride LZ + mvm= ↓ 33% DPNSS, placebo= ↓ 4% DPNSS	[103]

Abbreviation: BCVA: best corrected visual acuity, CS: contrast sensitivity, DPNSSs: diabetic peripheral neuropathy symptom scores, GS: glare sensitivity, hsCRP: high-sensitivity c reactive protein, LDL-C: low-density lipoprotein cholesterol, HDL-C: high-density lipoprotein cholesterol, LogMAR: logarithm of the minimum angle of resolution, LMZ: lutein, meso-zeaxanthin and zeaxanthin, MPOD: macula pigment optical density, mvm: multivitamins and mineral, OD: oculus dexter, OS: oculus sinister, RRD: retinal response density, VA: visual acuity

T: treatment, U: untreated, C: control, ↑: increased, ↓: decreased, d: day, m: month, y: year

### Anti-oxidative stress benefits of macular carotenoids

Oxidative stress is a primary and early driver of retinal damage in retinopathy. Antioxidative therapies often target foundational protective effects by neutralizing reactive oxygen species (ROS) and preventing initial cellular injury. These ROS are by-products of oxidative stress occurring in the mitochondria through glucose metabolism and auto-oxidation, and they are the major effectors leading to DR initiation and progression via the accumulation of superoxide ions, hydrogen peroxide, and hydroxyl radicals in the cell. In an event where the ROS becomes imbalanced, the polyol, hexosamine, protein kinase C (PKC) and growth factors are activated in the cell and this triggers other signalling pathways that promote oxidative damage to DNA, lipid membranes and proteins that protect the cell [108]. The antioxidant scavenging compounds that correct this imbalance are either endogenous enzymes produced by the body like superoxide dismutase (SOD), glutathione peroxidase (GPx), catalase (CAT), cytochrome oxidase, and or non-enzymes like glutathione (GSH), melatonin, or exogenous herbal-based compounds like carotenoids.

Based on animal trials (Table 5) and cellular studies (Table 6), lutein showed protection against oxidative stress. These studies all used purified commercially available lutein with significant reductions in key oxidative pathways in 90% of the studies. Some exert multiple benefits including protective effects during later pathological stages of disease.

**Table 5:** Animal trials using commercially available lutein (L) oral supplementation as an early retinopathy remedy acting against angiogenesis, apoptosis, inflammation, and oxidative stress

Lutein source (% purity)	Experimental model stressor	Dosage	Outcome of lutein treatment	References
Commercial L	C57BL/6 mice. **S** = STZ-induced, 60 mg/kg for 3d	**T**: 0.1% (wt/wt) L, **U**: DR stressed, **C**: healthy mice. Orally for 4m	↓ superoxide anions. ↓ ERK activation. ↑ BDNF, synaptophysin. ↓ cell loss in INL, IPL, + GCL of retina. Prevented ↓ of visual impairment compared to untreated DR	[109]
Commercial L (70)	Male albino mice. **S** = alloxan-induced 200 mg/kg	**T1**: 0.2 mg/kg L, **T2**: (L + insulin (500 mU/g), **U**: normal + DR stressed, **C**: healthy mice (citrate buffer) + DR (500 mU/g insulin). ig for 14d	Blood glucose same in untreated DR mice compare to DR treated with L, but insulin treated DR ↓ blood glucose. ↓ MDA, ↑ GSH, + GPx activity. ↑ ERG b-wave amplitude compared to untreated DR. ↓ NFκB compared to untreated DR. L + insulin has significant effect than each separately	[110]
Commercial L	Male albino mice. **S** = alloxan-induced 200 mg/kg	**T**: 100 mg/kg L, **U**: normal + DR stressed, **C**: healthy rat + DR with ebselen. Orally for 3d	↓ MDA. ↑ GSH compared to the untreated DR	[111]
Commercial L	Male Wistar rats. **S** = STZ-induced 65 mg/kg	**T**: 0.5 mg/kg L, **U**: normal + DR stressed, **C**: healthy rat + DR with 13.3 mg/kg DHA, + 50 mUI/g insulin. Orally for 12w	↓ MDA. ↑ GSH + GPx activity. ↓ nitrotyrosine levels. ↓ caspase-3 + TUNEL-positive cells compared to untreated DR. ↑ ERG b-wave amplitude Retinal layer not affected compared to the untreated DR.	[112]
Commercial L (10)	C57BL/6J Ins2^Akita/+^. **S** = heterozygote mice	**T**: 2.1, 4.2 + 8.4 mg/kg/d L, **U**: genetic DR model, **C**: healthy mice (water). Orally for 4.5m, 6.5m + 9m	Effective = 4.2 + 8.4 mg/kg ↓ Iba-1 + CD68 expression compared to the untreated DR (4.5m). ↓ VEGF in the retina of DR compared to the untreated DR (6.5m + 9m). ↓ vascular leakage in DR treated compared with untreated DR (6.5m + 9m). ↑ occludin in treated DR compared to the untreated DR (6.5m + 9m). ↑ ERG scotopic a-wave + b-wave amplitude in treated DR compared to the untreated DR (6.5m + 9m)	[113]
Commercial L	Female Wistar rats. **S** = STZ-induced 55 mg/ kg	**T1**: 0.5 mg/kg/d L, T2: 0.6 + 3.0 mg/kg/d AST, **U**: DR stressed, **C**: healthy rat with citrate buffer. ig for 8w	Effective = L (0.5 mg/kg), AST (0.6 + 3.0 mg/kg) ↑ ERG b-wave amplitude, compared to untreated DR. ↓ 8-OHdG, nitrotyrosine, but not acrolein in the L-treated, and 0.6 + 3.0 mg/kg/d AST treatment ↓ 8-OHdG, nitrotyrosine + acrolein. ↓ ICAM-1, MCP-1, + FKN compared to the untreated DR (protein and mRNA). ↑ HO-1 + PRDX compared to untreated DR No treatment affected Trx (mRNA). ↓ overexpressed NF-κB–DNA binding activity ↓ total retina thickness.	[114]
Mixed micellar L (> 97)	Male Wistar rats. **S** = STZ-induced 36 mg/kg	**T**: 39 nmol L, **U**: DR stressed, **C**: healthy rat with citrate buffer. Orally for 8w	↓ MDA + carbonyl expression (serum + retina). ↑ serum GSH (83%) compared to the untreated DR. ↓VEGF, VEGFR2, Nrp1, Xbp1 + Hif1α compared to untreated DR. ↓ cellular loss of the histological layers. ↑ SOD2	[115]
Marigold petal L (99)	Swiss albino mice. **S** = LPS-induced 3 mg/kg	**T1**: 200 µM micellar L, **T2**: 200 µM + PL with L + 200 µM -PL without L, **U**: DR stressed, **C**: healthy mice with saline. Orally for 15d	Effective = micellar L, +PL, + -PL (All T) All T ↓ NO, but + PL had the highest. All T ↓ MDA, but + PL had highest compared to the untreated DR (serum + retina). (+ PL) ↓ protein carbonyl = 74.6% in serum + 75.6% in retina. +PL: -PL antioxidant= ↑ SOD (15.4:17.1%), ↑ CAT (17.3:17.2%), ↑ GPx (16.1:15.0%), ↑ GR (17.7:21.8%), + ↑ GSH (20.1:18.7%). +PL= ↓ TNF-α, IL-6, MCP-1, + IL-1B compared to untreated DR. ↓ PGE2 = micellar (42.4%), +PL (78.4%), + -PL (64.6%) compared to untreated DR. ↓ NF-*κ*B p65 = + PL (43.2%), -PL (64%), micellar L (78.4%) compared to untreated DR. +PL= ↓ COX-2 + iNOS expression. +PL= ↓ NF-κB p65 activity	[116]
Commercial L	Male albino Wistar rats. **S** = cisplatin-induced 5 mg/kg, another 2.5 mg/kg after 1h	**T**: 0.5 mg/kg/d L, **U**: DR stressed, **C**: healthy rat with only sunflower oil. Orally for 14d	↓ MDA + ↑ GSH. ↓ IL-1β + TNF-α levels compared to the untreated DR. Prevent retinal damage on histological layers	[117]
Commercial L	Male Wistar rats. **S** = STZ-induced, 36 mg/ kg	**T**: 10 µM L, + 5, 10 µM lactucaxanthin (lac), **U**: DR stressed, **C**: healthy rat with citrate buffer. Orally for 8w	Effective = L + lac (all T) ↓ FBG compared to the untreated DR. Lac T= ↓ HIF-1α; 1.34-fold + VEGF; 1.07-fold. L= ↓ HIF-1α; 1.58-fold + VEGF; 1.68-fold. Both alter retinal damage	[118]
Commercial L	Male C57BL/KsJ-db/db mice. **S** = hyperoxia-induced, 100% O_2_ + flicker light-induced stress, 30 lux/12 Hz frequency	**T**: 13.3 mg/kg/d L, + 133.3 mg/kg/d *Trapa bispinosa* (TBE), **U**: DR stressed, **C**: healthy mice with placebo. Orally for 14w	Effective = L 13.3 mg/kg/d + TBE 133.3 mg/kg/d ↑ retinal blood flow (50%) compared to the normal control after 14w. ↓ GFAP + VEGF expression in DR compared to untreated DR + control.	[119]

Abbreviations: ALR: aldose reductase, Bax: bcl-2 Associated X-protein, BDNF: brain-derived neurotrophic factor, BRB: blood retinal barrier, CAT: catalase, CD68: a cluster of differentiation 68, Cl: Clearance, DHA: docosahexanoic acid, egr1: early growth response 1, ERK: extracellular signal-regulated kinase, EYA3: expression of eyes absent, FBG: fasting blood glucose, FKN: fractalkine, GFAP: glial fibrillary acidic protein, HO-1: heme oxygenase-1, Iba-1: ionized calcium-binding adapter molecule 1, ICAM-1: intercellular adhesion molecule-1, IL-1β: interleukins 1 beta, INL: inner nuclear layer cells, iNOS: inducible nitric oxide synthase, LDH: lactate dehydrogenase, LNC: chitosan-sodium alginate lutein-loaded nanocarrier system, MCP-1: monocyte chemoattractant protein-1, MCP1: monocyte chemotactic protein-1, Bcl2: b-cell lymphoma protein 2, MDA: malondialdehyde, MRT: mean resident time, NF-κB: nuclear factor-kappa B, NGF: nerve growth factor, GSH: glutathione, p38 MAPK: p38 mitogen-activated protein kinases, PBS: phosphate buffered saline, PEDF: pigment epithelium-derived factor, PL: phospholipid nano-carrier, PRDX: peroxiredoxin, SDH: sorbitol dehydrogenase, SOD: superoxide dismutase, STAT3: signal transducer and activator of transcription 3, T_1/2_: half-life, Thy1: thymocyte antigen 1, TNF-α: tumour necrosis factors alpha, TUNEL: terminal deoxynucleotidyl transferase (tdt) dutp nick-end labeling, VEGF: vascular endothelial growth factor

T: treatment, U: untreated, C: control, ↑: increased, ↓: decreased, h: hour, m: month, d: day, w: week, ig: intragastric tube

**Table 6:** In-vitro cellular trials using lutein as an early retinopathy remedy acting against angiogenesis, apoptosis, inflammation, and oxidative stress

Lutein source (%purity)	Stressor	Dosage	Outcome of treatment	References
Commercial L	30 µM glucose for 48h	**T1**: 10 µM L, **T2**: 5 + 10 µM Lac, **U**: glucose stress, **C**: 0.01% of DMSO for 48h	Effective = Lac; 5 µM + 10µM, L; 10 µM. T1-T2 ↑ ZO-1 expression = Lac; 5 µM (0.73-fold), + Lac; 10 µM (0.62-fold), + L; 10 µM (0.66-fold), T1-T2 ↑ occludin expression = Lac; 5 µM (0.89-fold), + Lac; 10 µM (0.95-fold), + L; 10 µM (1.00-fold), T1-T2 revert cell migration = Lac; 5 µM (114.49%) + Lac; 10 µM (92.75%), + L; 10 µM (128.98%) T1-T2 ↓ cellular ROS = Lac; 5 µM (1.30-fold) + Lac; 10 µM (0.97-fold), + L; 10 µM (1.90-fold). Attenuated Ψm by ↑ cellular aggregate = Lac; 5 µM (1.15-fold) + 10 µM (1.60-fold), + L; 10 µM (1.82-fold). T1-T2 ↓ MDA = Lac; 5 µM (65%), Lac 10 µM (59%) + L; 10 µM (65%). T1-T2 ↑ SOD (3.2-fold), GR (2.0-fold), CAT (1.6-fold) = Lac; 5 µM. T1-T2 ↑ SOD (5.6-fold), GR (2.5-fold), CAT (2.2-fold) = Lac; 10 µM. T1-T2 ↑ SOD (3.3-fold), GR (2.6-fold), CAT (2.2-fold) = L; 10 µM. T1-T2 ↓ HIF-1α = Lac; 5 µM (0.94-fold), + Lac; 10 µM (0.81-fold) + L; 10 µM (1.07-fold). T2 ↓ XBP1 (1.42-fold), ATF4 (1.21-fold), ATF6 (0.88-fold) = Lac 5 µM; ↓ XBP1 (0.86-fold), ATF4 (1.42-fold), ATF6 (1.00-fold) = Lac; 10 µM, T1 ↓ XBP1 (1.64-fold), ATF4 (1.10-fold), ATF6 (1.08-fold) = L; 10 µM T1-T2 ↓ VEGF = Lac; 5 µM (0.97), + Lac; 10 µM (0.71-fold) + L; 10 µM (1.02-fold). T1-T2 protect retinopathy compared to untreated glucose stressed	[118]
Commercial L	5.6 mM low glucose, or 30 mM high glucose for 24h	**T**: 0.01–2 µg/mL L, **U**: glucose stressed for 24h	Effective = L 2 µg/mL. ↓ SA-β-gal activity. ↓ ROS activity. ↑ SIRT1 expression (protein, + mRNA)	[120]
Wolfberry L	36 mM glucose for 48h	**T1**: 0.625 + 1.25 µM L, **T2**: 0.625 + 1.25 µM Z, **U**: glucose stressed, **C**: Mannitol + PBS for 48h	T1-T2 ↓ ROS activity. T1-T2 ↑ FOXO3α, SOD, and thioredoxin activity. Activate the AMPK signaling pathway	[121]
Commercial L (10)	TBHP-induced stress, DMSO in tetrahydrofuran (2:1)	**T**: 0.5, 1 + 2 µM L, **U**: TBHP stressed, **C**: DMSO for 36h	Effective = 1 µM. ↓ BCO1 + SR-B1, ↑ BCO2, LDLR, VEGF expression pattern. CD36 was not affected compared to the untreated control. ↓ cell viability (15%), compared with that in the normoxic group. ↓ tBHP-induced cell death compared to untreated cells.	[122]
Commercial L (95)	Light stress by exposure to 0.46 mW/cm2 fluorescent light in presence of rose bengal for 0.33, 0.5, 0.67 + 1h	**T1**: 10–40 µM L, **T2**: 10–40 µM Z, **T3**: 10–40 µM dehydrolutein, **U**: fluorescent light stressed, **C**: 0.2% DMSO for 19d	Effective = L + Z; 20, 40 µM for photooxidation. Quenching rate constant = L (0.55) × 10^10^ M^− 1^s^− 1^_,_ + Z (1.2) × 10^10^ M^− 1^s^− 1^, + dehydrolutein 0.77 × 10^10^ M^− 1^s^− 1^. T1-T3 ↓ O_2_ consumption rate = L (68%), Z (75%) + dehydrolutein (72%) at 20 µM. T1-T3 ↓ photo-damage = L (92%), Z (90%) + dehydrolutein (95%) in 0.33h light exposure; L (88%), Z (86%) + dehydrolutein (87%) in 0.5h light exposure; L (67%), Z (66%) + dehydrolutein (64%) in 0.67h light exposure; L (41%), Z (38%) + dehydrolutein (40%) in 1h light exposure.	[123]
*Chenopodium album* L (≥ 95)	20 + 30 µM glucose for 24h	**T**: 0-2.5 µM L. **U**: glucose stressed. **C**: DMSO for 24h	Effective = L; 0.5 + 1 µM. Not toxic to cell viability. ↓ MDA + protein carbonyl = L 1 µM. Restores ΔΨm expression = L 1 µM. ↑ SOD2 (39%) + CAT (32%). ↑ HO-1 + GSH = L 1 µM. ↑ AKT + ERK = L 1 µM. ↓ phosphorylated p-38 ↑ localization of Nrf2 in the nucleus.	[124]
Commercial L	7.5 mM glucose for 24h	**T**: 1 µM L. **U**: glucose stressed. **C**: 100 mM tunicamycin for 3h	↑ BiP (100%). ↓ unspliced XBP1 (mRNA). ↑ spliced XBP1 + p-IRE1 expression. ↑ translocation of ATF4 (mRNA), ATF4 expression (protein), + ↑ CHOP expression. ↑ HERP, TRB3, HRD1, p58IPK, EDEM, + PDI expression compared to the untreated stressed cell.	[125]

Abbreviations: Akt: protein kinase b, ATF4: activating transcription factor 4, ATF6: activating transcription factor 6, BCO1/2: β-carotene 15,15’-oxygenase 1/2, BiP: binding immunoglobulin protein, CAT: catalase, CHOP: c/ebp-homologous protein, DMSO: dimethyl sulfoxide, EDEM: er degradation enhancing alpha-mannosidase like protein, ERK: extracellular signal-regulated kinase, FOXO3α: forkhead O transcription factor 3α, GP: glutathione peroxidase, GSH: glutathione, HERP: homocysteine inducible ER protein, HO-1: heme oxygenase-1, HRD1: erad-associated e3 ubiquitin-protein ligase hrd1, Lac: lactucaxanthin, LDLR: low-density lipoprotein receptor, LNC: lutein-loaded nanocarrier system, MDA: malondialdehyde, O_2_: oxygen, p58^IPK^: protein kinase inhibitor p58, PBS: phosphate buffer saline, PDI: protein disulfide isomerase, p-IRE1: phospho-inositol requiring enzyme 1, ROS: reactive oxidative species, SOD: super-oxide dismutase, TBHP: *tert*-butyl hydroperoxide, TRB3: tribbles pseudokinase 3, VEGF: vascular endothelial growth factor, XBP1: x-box binding protein 1 spliced, ΔΨm: change in mitochondrial membrane potential

T: treatment, U: untreated, C: control, ↑: increases, ↓: decreases, h: hour, d: day, m: month, y: year

Lutein protects against oxidative stress by promoting the antioxidant enzymes (CAT, GSH, GR, SOD, GPx) against reactive intermediates. According to Miranda et al. (2006), the plasma glycaemic level in normal mice was like diabetic mice stress induced with 200 mg alloxan, but diabetic stress elevated MDA levels because of lipid peroxidation that occur in the hyperglycaemic state of the cell, and this downregulated the endogenous antioxidant, GSH. The imbalance in the redox haemostasis caused superoxide (O^−^ _2_) and peroxynitrite production, that was reversed by lutein like insulin treatments [111]. Similarly, the stressor effect of 5000 lx blue light in mice initiated severe ROS production, detected by the upregulation of protein markers (glucose-regulated protein; GRP78, activating transcription factor 6; ATF6, phosphorylated protein kinase RNA-like endoplasmic reticulum kinase; p-PERK and activating transcription factor 4; ATF4) associated with endoplasmic reticulum stress (ERS) causing photoreceptor damage in PED [126]. However, lutein and zeaxanthin blocked the production of ROS that caused retina damage through the activation of nuclear factor erythroid-derived 2-like 2 (Nrf2) expression and downregulation of phosphorylated c-Jun N-terminal kinase (p-JNK). Furthermore, lutein and zeaxanthin protection was supported by the scarce presence of TUNEL-positive cells, as this was an indicator of apoptotic damage within the outer nuclear layers (ONL) of the retina [126].

In a meso-zeaxanthin supplementation study, there were no measurable meso-zeaxanthin in controls and high fat induced DR rats before treatment. After mezo-zeaxanthin (100 mg/kg/d) administration, the meso-zeaxanthin level in the serum was elevated in the control, and high fat induced DR rats. The meso-zeaxanthin treatment restored the activity of SOD and CAT to minimize oxidative stress retinal damage compared to the untreated high fat induced DR rats. Furthermore, there was coordinated suppression of modulators of inflammation proteins (VEGF, iNOS, ICAM, NF-κB), and activation of oxidative stress genes Nrf2 and HO-1 [127].

In addition, most animal studies used oral administration for lutein and zeaxanthin by mixing with the chow diet or gavage, the efficacy can be affected by high enzymatic degradation, and strong gastric acid pH in the stomach. This digestive interference can be bypassed via the intragastric (ig) route for the administration of lutein (0.5 mg/kg/d) and astaxanthin (0.6 + 3.0 mg/kg/d) that reduced enzymatic degradation and strong gastric acid pH effects. This administration route was effective in treating the oxidative stress mediators (8-hydroxy-2’-deoxyguanosine; 8-OHdG, nitrotyrosine, and acrolein) elevated in the retina by peroxidation chain reaction of polyunsaturated fatty acids (PUFA) leading to oxidized lipid by-products generation from STZ-induction. Upon lutein administration by ig, there were reductions in 8-OHdG, nitrotyrosine and acrolein expression in the retina. This observation was linked to a restoration of homeostatic balance by increases of other antioxidant defence enzymes (hemeoxygenase-1; HO-1, and peroxiredoxin; PRDX) in the retina [114]. Hence, this suggested that the improved efficacy of macular carotenoids for retinopathy depended on the administration route to prevent enzymatic degradation and bind with specific transport proteins necessary for efficient delivery to the retina. Compounded differences in dosage (2.2–100 mg/kg/d for lutein, 2.2–60 mg/kg/d for zeaxanthin, and 2.1–100 mg/kg/d for combinations of both pigments) make it difficult to compare the effectiveness [76].

### Anti-inflammatory benefits of macular carotenoids

Inflammation is closely linked to oxidative stress and further propagates retinal damage. Blocking inflammation helps break this cycle and reduce tissue injury. Ocular inflammation occurs as a coordinated response of the immune cells, endothelial cells, and neurons to protect the eye by activation of mediators like cytokines, chemokines and adhesion molecules that can contribute to retinal damage [128]. When the protective integrity of the eye is breached by imbalanced redox haemostasis from ROS, various retinal cell types such as microglial, Müller glia, and astrocytes activate the release of inflammatory mediators [128]. Some proinflammatory markers in the serum, plasma and aqueous/vitreous humor associated with inflammation-induced retinopathy includes interleukins (IL-2/4/5/6/8/10, and interferon-γ), complement system (C2/3, and CFH), CC-motif chemokine receptor (CCR3), CC-motif chemokine ligand (CCL3, CCL5, CCL21), chemotactic protein 1 (MCP-1), macrophage inflammatory protein-1 alpha/beta (MIP-1α/β), stromal cell-derived factor 1 (SDF-1), ICAM-1, VCAM-1, TNF-α, C-reactive Protein (CRP), cyclooxygenase 2 (COX-2) [108, 128, 129]. The accumulative effect of these mediators causes chronic infiltration that compromise the cellular barrier defence, thereby aggravating retinal vascular permeability, vasodilation, and retinal thickening. This caused degeneration and reduction of cell numbers in the retinal histological layers, with the presence of atrophy, edema and detachment of the RPE layer from the choroid [117]. The treatment of lutein (0.5 mg/kg) reversed this inflammatory damage compared to untreated (Table 5). This retinal inflammation damage is linked to the elevation of VEGF in early DR of Ins2^Akita/+^ mouse, and reductions of occludin, showed that long-term (9 month) administration of lutein supplements significantly protected the retinal vasculature and preserved retinal functions compared to untreated DR [113]. In addition, photooxidative damage of RPE induce inflammation via the expression of complementary factor H (CFH), MCP-1, and IL8, that lutein and zeaxanthin treatment can restore by regulating harmful protein degradation [130].

### Anti-apoptotic benefits of macular carotenoids

Oxidative stress and inflammation can trigger programmed cell death of retinal cells, known as apoptosis. Key apoptosis pathways are implicated in retinopathy [131, 132], that are also common in other chronic diseases based on animal trials (Table 5) and cellular studies (Table 6). Dysregulation of these apoptotic proteins promotes retinopathy progression when the retinal cells are exposed to hyperglycaemia, with the ganglion cell layer (GCL) of the retina being the first and most vulnerable for apoptotic-induced neurodegeneration damage [131]. Hence, these apoptotic proteins serve as common biomarkers in the development of neural degeneration and vascular damage during accelerated accumulation of ROS, which further compromises the retina [133].

Lutein and zeaxanthin were beneficial in the reduction of apoptosis by repairing retinal damages and reducing cell senescence. In retinal pigment epithelial cells (ARPE-19), zeaxanthin promoted nuclear translocation of Nrf2 and induced the expression of phase 2 detoxification enzymes like heme oxygenease-1 (HO-1), NAD(P)H: quinone oxidoreductase (NQO-1), and g-glutamyl-cysteine ligase (GCL). GCL regulated the synthesis of GSH, an endogenous antioxidant able to scavenge ROS insult in the cell [134]. Zeaxanthin also protected the retina against tert-butyl hydroperoxide (t-BHP) induced mitochondrial dysfunction and apoptosis by reversing the cellular antioxidative capacity of copper-zinc superoxide dismutase (Cu-Zn SOD), glutathione peroxidase (GPx), and glutathione s-transferase (GST) in a rat model [134].

In response to oxidative stress from diabetes, forkhead O transcription factor 3α (FOXO3α) was activated, but with lutein and zeaxanthin treatment from wolfberry [121], phospho-FOXO3α, Ser253 and total FOXO3α expression was significantly restored in the retina and this prevented apoptosis. In addition, the stress biomarkers, binding immunoglobulin protein (BiP), protein kinase RNA-like ER kinase (PERK), activating transcription factor 6 (ATF6), and active caspase-12, were severely elevated in diabetic db/db mice but reversed by 1% wolfberry.

Other studies showed that diabetes induction in 4-month-old mice caused upregulation of caspase-3 in the GCL but not in the INL of the retina. This apoptotic marker in the GCL indicated neurodegeneration prior to vascular changes observed in early retinopathy [109]. This was supported by the suppression of neurotrophic proteins; brain-derived neurotrophic factor (BDNF), which was essential in the survival of inner retinal cells for normal vision. The impairment of BDNF levels in the retina neural layer resulted in function loss and can cause blindness. However, with a constant intake of lutein, caspase-3 activity was suppressed, and neurotrophic BDNF protein activation was restored. With this change, the apoptotic pathways attributed to retinopathy progression was prevented [109]. In other contexts, lutein and zeaxanthin reversed photoreceptor damage by more than 50% from peroxides and paraquat generated oxidative-induced stress, by attenuating the changes in mitochondrial membrane potential (ΔΨm) to prevent the release of cytochrome c which can aggravate apoptotic damage to the photoreceptors [135].

### Anti-angiogenesis benefits of macular carotenoids

Angiogenesis is a later-stage pathological process in retinopathy (especially diabetic and proliferative forms) by causing abnormal blood vessel growth and the vision-threatening complications of advanced diseases. Lutein and zeaxanthin can reverse the effects of angiogenic proteins implicated in endothelial cell membrane (ECM) breakdown, a feature peculiar to retinopathy. In the advent of retinal neovascularisation, the destruction of the basement membranes around the microvascular endothelium caused migration of endothelial cells, and the pericyte begins to proliferate into the surrounding matrix by the aid of integrins (adhesion molecule) to form abnormal neovessels [136]. This damage was accompanied by the loss of tight junction proteins in the inner and outer BRB, which increased vascular permeability thereby causing the release of proteins like growth factors, cytokines and chemokines which are mediators of angiogenesis. The imbalance from this cellular disruption promoted pro-angiogenic and anti-angiogenic signalling pathway proteins like erythropoietin (EPO), growth factors and their receptors including basic fibroblast growth factor (BFGF), connective tissue growth factor (CCN2/CTGF) [137], epidermal growth factor (EGF), hematopoietic growth factor (HGF), insulin-like growth factor (IGF), platelet-derived growth factor (PDGF), transforming growth factor-β (TGF-β) [138], vascular endothelial growth factors (VEGF) [108]. Furthermore, VEGF has also been associated with NADPH oxidase (Nox4) in the retina, to elevate ROS pathologic angiogenesis in oxygen-induced retinopathy mice [139].

Earlier studies on lutein (39 nmol) remedy for STZ-diabetic mice, showed decreased VEGF and its receptor by 5-fold and 0.8-fold at the mRNA and protein level, respectively, at the end of 8 weeks treatment [115]. Consequently, the SOD was elevated by 0.7-fold to confer antioxidative protection on the mice retina after treatment compared to the untreated DR. This showed the connection between the angiogenic and oxidative mechanisms to the development of DR [115]. The suppression of VEGF also directly impacted the downregulation of the hypoxia-inducible factor 1α (HIF-1α), and X-Box binding protein 1 (Xbp1) which were the hypoxia genes co-expressed in DR [115]. A daily administration of lutein (4.2 mg/kg and 8.4 mg/kg) for 9 months revealed a downregulation of the VEGF protein compared to the untreated DR in Ins2^Akita/+^ mouse retina, to restore the tight junction protein; occludin, which protected the retinal vascular integrity [113]. In another study by Anitha et al. (2021), lutein (10 µM) treatment on diabetic mice, showed downregulation of HIF-1α (1.58-fold) and VEGF (1.68-fold) expression. Angiogenic cellular damage by endoplasmic reticulum stress (ERS) proteins like XBP1, ATF4 and ATF6 were upregulated in DR, but with lutein treatment, these markers were supressed by 1.64-fold, 1.10-fold, and 1.08-fold, respectively, that was additionally supported with histological restoration of ONL, INL, and RPE damaged by hyperglycaemia [118].

Furthermore, the effect of the VEGF protein secretion revealed an association with NADPH oxidase (Nox4) in the retina, which was responsible for ROS generation that caused angiogenesis in oxygen-induced retinopathy mice [139]. In VEGF-induced retinal neovascularisation peculiar to PDR and AMD, lutein and zeaxanthin showed independent attenuation of angiogenesis contributing to DR development. Lutein and zeaxanthin (0.05–1 µg/µL) were biocompatible maintaining high cell viability of human retinal microvascular endothelial cells (RMVEC), compared to anti-VEGF antibody drug treatments like bevacizumab, which exhibited a significant loss of cell viability by 62% with 0.5 µg/µL treatment and 52% with 1 µg/µL treatment [140]. Also, the combination of both lutein and zeaxanthin at a 5:1 ratio, as a commercially available supplement (Macushield) [98], further improved their bioavailability in the plasma with increases from 0.372 to 3.163 µg/dL for lutein and 0.117 to 0.391 µg/dL for zeaxanthin following 12 weeks daily supplementation [141].

## Polymers used as adjuvants in ocular delivery suitable for early retinopathy remedy

The benefits of macular carotenoids from diet and supplementation via oral delivery varied greatly based on multiple factors in the gastrointestinal tract, and this can affect the minimal effective dose to exert quantifiable or physical benefits. Because of this, ocular drug delivery was developed for direct contact to the eye and closer proximity to the posterior eye [142]. Ocular applications of drugs can be in the form of topical liquids (eye drops and suspensions), or semi-solids (gels and ointments), that are more suitable for anterior eye diseases (AED) due to the natural barriers of the eye [143] that restrict drug penetration to the posterior, where most PED visual aberration originates. Therefore, ocular deliveries by topical liquid and semi-solid forms raise challenges like high tear clearance of the drug, short resident time, poor bioavailability (< 5%) and increased instability depending on the drug. This barrier also limits the absorption of drugs to penetrate the eye, and to bypass this, injections of the intravitreal, subretinal, and suprachoroidal are currently used for the administration of drugs like anti-VEGF and corticosteroids [144, 145]. Intraocular injections elicit rapid pharmacological responses in < 7 days [146], with high dosing accuracy, but is invasive which can lead to deterioration of the eye like retinal detachment, requires frequent expert administration that can be inaccessible, is costly, and incur low compliance by patients with needle phobia [143].

**Fig. 4:**
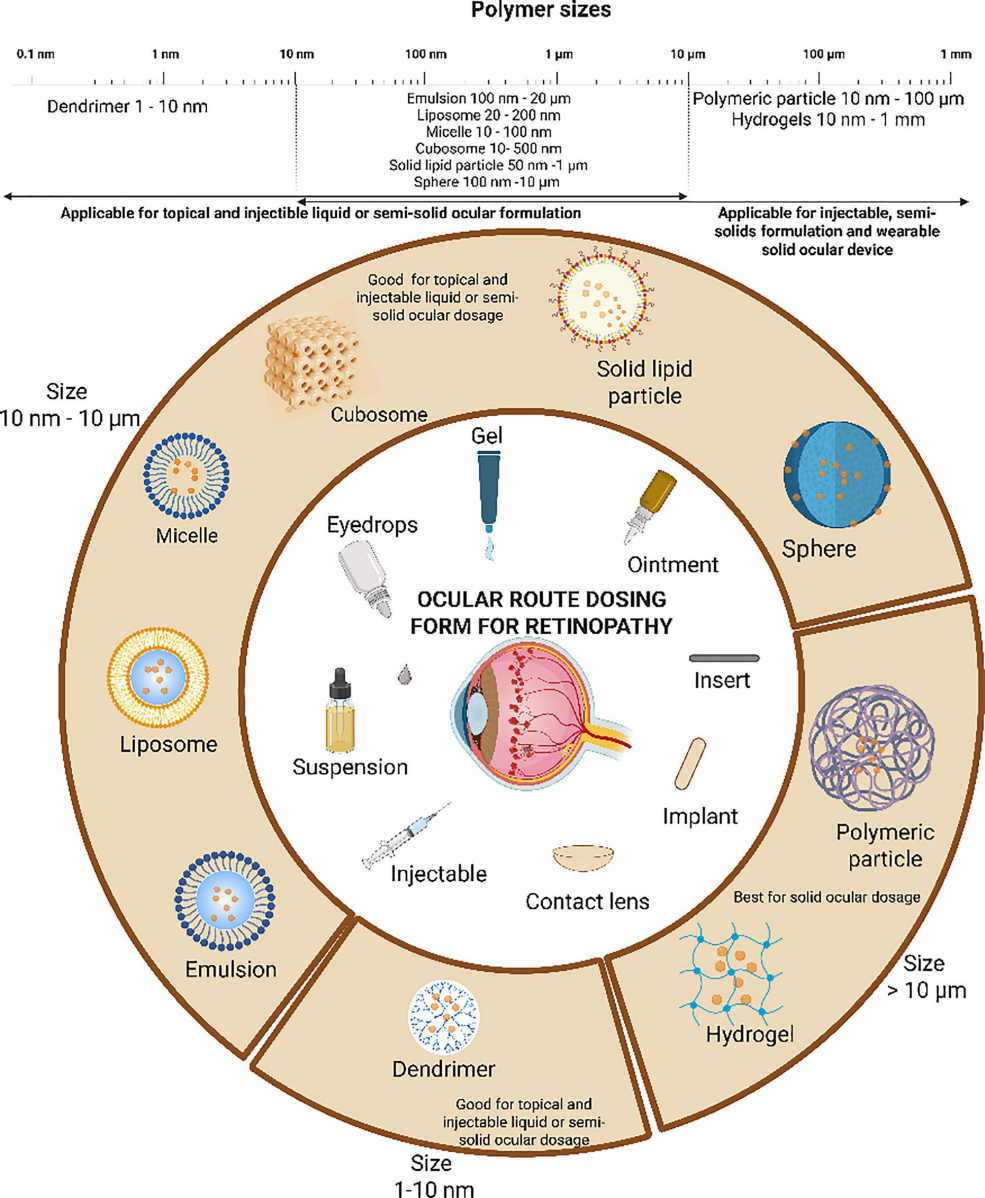
Different sized polymers used for the encapsulation of herbal-based compounds (outer circle) and the ocular routes of delivery (inner circle). Note: Orange dot on images represented herbal-based compounds. Created with Biorender

The introduction of innovative polymers (dendrimers, hydrogels, liposome, micelles, polymeric particles, emulsion) with diverse size variations ≥ 1 nm ≤ 1000 μm (nano and microscale) can be used as adjuvants to improve ocular delivery (Fig. 4). These polymers function to encapsulate phytochemicals in liquids (eye drop and suspension), semi-solids (ointment and gel), solids (insert, implant, and contact lens) and mixed dosage to protect the phytochemicals and make them more stable in formulations resulting in longer activity in the eye. Additionally, this reduces the need for multiple applications, overdosing and therefore more effective for longer periods with slow release in the eye as a low dose medication [3, 147]. Administering lower doses of medication decreases the risk of overdose or hyper-reactivity due to immunologic or inflammatory reactions, particularly in individuals who have heightened sensitivity to the drug [148]. Encapsulations of nanoparticles (NP; <200 nm) and microparticles (> 1000 nm) can be constructed by self-assembly materials to manifest a unique continuous microstructure in spherical, cubic, chain-like and star architecture. The plasticity of these NP makes it suitable to be stratified into forms embedded with natural and synthetic polymers such as liposomes.

Liposomes are a subset of particles composed of an aqueous core surrounded by a lipid bilayer to form spherical vesicles. The size of liposomes (20–200 nm) can be tailored for specific delivery of both water-soluble or non-soluble molecules, and this depended on the aggregate of lipids used in the preparation. Liposomes can be modifiable for the delivery of high drug payloads, and its biocompatibility influenced their biological applications.

Other forms of NP include solid lipid particles that are nanosized (50–1000 nm) but are different from nanoliposomes due to the solid state of lipids at room temperature. It had emulsifiers which stabilized the structures when it became aqueous, thereby protecting the integrity of the compounds in it. In comparison to other NP systems, the composition of solid lipid particles enhanced their physical stability for increased drug solubility, controlled release and ease of large-scale production [3].

Micelles are another subset of particles made up of amphiphilic polymers characterised by a hydrophobic core and a hydrophilic shell, with the size ranging between 5 and 100 nm. This feature made micelles selectively suitable for poor water-soluble drugs and lipophilic cell targets. Cubosome are another particle made up of continuous cubic liquid crystalline carriers of 10–500 nm size constructed by emulsification of lipids in water and structured in 3D resembling honeycombs. The cubosome serve as adjuvants for a wide range of phytochemicals, peptides, and nucleic acid due to their large surface area. They are easy to prepare, biodegradable, and safe [143].

The solid sphere is another uniformly distributed solid spherical particle composed of materials like polymers, metals, or silica. It has a broad size range of 100 nm to 10 μm and can be engineered as a carrier for a diverse range of compounds, which allowed them to reach specific targets. The solid sphere has a high surface area to volume ratio that made it a reactive and highly interactive particle with compounds within or outside its surface [143].

Dendrimers are another subset of particles characterised by its highly branched star and tress shaped 3D structure. Its shape has a central core, inner branch shells, and a functional group in the outer shell that made it attractive for delivery of both hydrophilic and lipophilic drugs [143]. Dendrimers are used in drug delivery, gene therapy and in chemical catalysis as sensors due to its high surface area and the functional group tagging. The size is small, ranging 1–10 nm, but compared to the other NPs, it is more complex, expensive to synthesise and may exhibit toxicity depending on the functional group present [149].

Lastly, hydrogels are composed of monomeric units of polymers physically, chemically, or enzymatically crosslinked to form a 3D complex network with sizes between 10 nm and 1 mm that are easily fabricated for solid dosage in ocular delivery (Fig. 4). The physicochemical properties (transparency, high absorption, and structurally flexible) of hydrogels made it suitable for diverse ophthalmic applications that includes wearable contact lens, implants, inserts, tissue repair and grafting [150]. Based on the exposed environment, hydrogels can shift responses to external stimuli like ion, light, pH, and temperature to regulate the drug release for prolonged duration. It has a better muco-adhesion and porosity to absorb high volumes of fluid than other NP, and this was suitable for usage as topical liquids, semi-solid, solid and mixed dosages for ocular, oral, nasal, or transdermal drug delivery [151]. The high hydrophilic property was vital to prevent tear clearance of drugs from the eye, and this improved contact time of the drug to the eye for bioavailability. In addition, the monomeric composition highly impacted the properties aiding its ability for different drug release matrixes by diffusion, swelling, and chemical-induction [151].

Each of these polymers can increase the efficacy of ocular treatments containing bioactive compounds for retinopathy in animal trials (Table 7) and cell studies (Table 8). The benefits in visual function, and the underlying mechanisms to alleviate oxidative stress, inflammation, apoptosis and angiogenesis provide strong support for their broader application to deliver adequate phytochemicals as suspensions or eyedrops, including lutein, for the management of various ocular diseases like cataracts [152], corneal permeation [153], AMD [154] and retinopathy [155, 156]. In addition, to prevent free carotenoid derived aldehydes (CDA) autooxidation [157], and instability during their release from dietary sources or supplements, carotenoids need to be esterified and encapsulated in different polymer matrixes to improve bioavailability and facilitate its administration at low doses for sustained release [154, 156]. The popular direction of polymers for PED remedy is still in experimentation but is pivoted towards the development of non-invasive wearable ocular devices (WOD) embedded with bioactive compounds that can be used for point-of-care application, to serve as a preventative early disease phase option.

**Table 7:** Polymer encapsulated phytochemicals used for in-vivo retinopathy remedy acting against angiogenesis, apoptosis, inflammation, and oxidative stress

Delivery polymer system (dosage form)	Encapsulation	Phytochemical	Experimental model stressor	Dose/model stressor	Effectiveness	References
Hydrogel (gel)	BSA, Pluronic F127, Pluronic F68 + glut	Curcumin (Cur)	New Zealand White rabbits of either sex. **S**: no stressor.	**T**: 50 µl Cur polymer. **C**: free cursus. Administered 50 µL in the conjunctival sac of right eye.	Cur content in AH=↑5.6-fold for T than control. Ocular bioavailability of cur in AH= ↑ 4.4-fold in T than control. T irritation score = 0 (non-irritant). T promoted recovery of syringe punctured eye; less swelling, corneal opacity + redness compared with free cursus	[158]
Liposome (eye drop)	Lecithin, cholesterol, + stearylamine	Naringenin (NAR)	Adult male New Zealand albino rabbits. **S**: 50 µL of 0.025 M α-AAA by iv	**T1**: 200, 500, + 800 µg/mL NAR liposome. **T2**: liposome only (placebo). **T3**: 1.25 mg/50 µL bevacizumab (drug). **U**: DR stress. **C**: unstressed rabbit no treatment. Topical suspension for 3w (2 dose daily for 5d weekly). Note: Left eye served as negative untreated control.	NAR size = 48–150 nm, stable for 3 m at room temperature. T1=↓clinical score of NV compared to T2. T1= ↓number of blood vessels. Microscopic structure improved better in 800 µg/mL like T3 and control. Reversed retinal damage by α-AAA with T1 800 µg/mL.	[159]
Micelles (eye drop)	VA64 = PVP + VA	Apocynin (APO)	Male BALB/c mice. **S**: 5 µL 0.45% BAC, 2 dose/d (9 am + 9 pm to the right eye) for 1-6d. From 7–13d, 5 µL 0.60% BAC 1 dose/d	**T1**: 5 mg/mL APO. **T2**: 90 mg/mL VA64. **T3**: 1 mg/mL APO-VA64. **T4**: 5 mg/mL APO-VA64. **C**: PBS (-ve), + 0.1% HA (+ ve) in unstressed mice. Administered 5 µL, 3 dose/d, for 7d.	Encapsulated APO storage stability = 94% at 4 °C + 93% at 25 °C in 12w. ↑ solubility of T1 = 0.26, 0.13, + 0.26 mg/mL, to T2-T3 = 69, 70, + 77 mg/mL in water, PBS, and artificial tears, respectively. T3 caused positive effect on the BAC-induced damage. T3 improved corneal opacity score (4.1), ocular surface lacrimal film (7.53%) + tear production (6.4 mm), similar for T4. T3-T4= ↓ HMGB1, IL-6, NF-κB, + TNF-α compared to the healthy control corneas. APO-VA64 was able to penetrate and uniformly distributed across the whole cornea	[160]
Polymeric particle (film)	PEO N10	Hesperetin (HT)	Healthy New Zealand white rabbits. **S**: no stressor.	**T1**: 10% (8 mg; dose: 0.8 mg) HT. **T2**: 20% (8 mg; dose: 1.6 mg) HT. **C**: PBS in unstressed rabbit. HT film was placed in the rabbit eye (conjunctival sacs). Both doses were checked for 1h + 3h, and the 20% dose was checked for 6h.	Value of HT at the back of eye for T1(10%) = VH; 0.05 µg/g + retina-choroid; 7.8 µg/g. Value of HT at the back of eye T2(20%) = VH; 1.4 µg/g + retina-choroid; 23.5 µg/g. Doubling the dose from T1(10%) to T2(20%) = ↑ conc. by 10-fold in scleral + 28-fold in VH after 1h. HT in T1(10%) conc. low in AH + retina-choroid at 3h = 1.2 + 1.8 µg/g compared to 1h = 4.2 + 7.8 µg/g. HT in T2(20%) conc. low in AH + retina-choroid at 3h = 3.4 + 3.3 µg/gm compared to 1h = 7.2 + 23.5 µg/gm. Value of HT in T2(20%) at 6h = VH; 0.03 µg/g, retina-choroid; 0.4 µg/g, iris ciliary bodies; 1.5 µg/g + sclera; 6.1 µg/g. No damage to the cornea for the T2(20%) film for 6h compared to control with corneal epithelium swelling	[161]
Polymeric hydrogel (eye drop)	PVP K-17PF	Kaempferol (KAE)	Healthy New Zealand white rabbits. **S**: 0.5% w/v sodium arachidonate in PBS (pH 7.4)	**T1**: 4.5 mg/mL free KAE. **T2**: 0.5, 1.5, 4.5 mg/mL 17PF-KAE. **C**: 1.0 mg/mL DIC in DR stress rabbit. Administered 50 µL each for 0.5h.	↑ T2 cellular level = 3.33-fold, 4.73-fold, 7.42-fold, 13.49-fold, + 12.47-fold at 0.08h, 0.17h, 0.33h, 0.5h + 1h incubation, respectively. T2 storage stability = 91% in 4 °C + 87% in 25 °C in 6w. ↑ permeability coefficient = 4 × 107 cm/s for T2, + 2 × 107 cm/s for free T1. Steady state flux = 2 µg/[cm2s] for T2, + 1 µg/ [cm2s] for T1. Absence of redness, inflammation, or increased tear production + satisfactory epithelium stroma in T2. T2= ↓ VEGF, CD54, IL-6, + TGF-β1 in the cornea	[162]
Polymeric hydrogel (suspension)	Chi + NaA	Lutein (L)	Healthy male Wistar rats. **S**: 38 mg/kg STZ single dose ip	**T**: 600 µM LNC. **U**: DR stressed. **C1**: unstressed rat with micellar L. **C2**: DR stressed with micellar L. Administered 0.2 mL orally for 16h.	T easily crossed the intestinal barrier + BRB. T ↑L in the plasma and retina compared to C1. Bioavailability of L from T = 739-fold. L in plasma= ↑ C_max_ 68 ng/mL; AUC 373 ng/mL in C1 to C_max_ 183 ng/mL (2.7-fold); AUC 1158 ng/mL (3.1-fold) in T after 16h. L in retina= ↑ C_max_ 12 ng/mL; AUC 106 ng/mL in C1 to 40 ng/mL (3.3-fold); AUC 554 ng/mL (5.2-fold) in T after 16h. L in plasma=↑ C_max_ 15 ng/mL; AUC 120 ng/mL in C2 to C_max_ 77 ng/mL; AUC 768 ng/mL in T after 16h, T in retina= ↑ C_max_ 7 ng/mL; AUC 569 ng/mL in C2 to C_max_ 29 ng/mL (4.0-fold); AUC 1588 ng/mL (2.8-fold) in T after 16h. T ↑ HDL + ↓ LDL compared to untreated DR	[156]
Polymeric hydrogel (eye drop)	PVP K-17PF	Naringenin (NAR)	Healthy New Zealand white rabbits. **S**: 0.5% sodium arachidonate in PBS (pH 7.4)	**T1**: 5 mg/mL free NAR. **T2**: 5 mg/mL NAR + 100 mg/mL 17PF (17PF-NAR). **C**: 0.1 mg/mL PBS in DR stress rabbit (-ve), + 5 mL:5 mg PBS: DIC in DR stress rabbit (+ ve). Administered 4 times (50 µL/drop) 0.17h apart.	NAR release in T1 = 88% + NAR release in T2 = 71% at 10h of incubation. T2 storage stability = 94% at 4 °C + 92% at 25 °C in 12w. Antioxidant activity higher in T2 = T2; 0.5551, T1; 0.2578 at 25 µg/mL in 5m. T2; 0.7217, T1; 0.3487 in 2h, T2; 1.0346, T1; 0.7947 at 125 µg/mL in 15m. T2 ↑ aqueous solubility of NAR by 921-fold. Absence of pathological changes in iris, retina, or sclera in T2. Good ocular tolerance.	[163]

Abbreviation: AH: aqueous humor, APO: Apocynin, AUC: area under the curve of plasma concentration as a function of time, BRB: blood retinal barrier, Chi: chitosan, C_max_: maximum plasma concentration, Conc: concentration, Cur: curcumin, Cursus: curcumin suspension in gel, DIC: diclofenac sodium, HA: sodium hyaluronate, HDL: high density lipoproteins, HT: Hesperetin, KAE: kaempferol, L: lutein, LDL: low-density lipoproteins, LNC: lutein loaded nanocarrier system, NaA: sodium alginate, NAR: naringenin, NV: neovascularisation, PBS: phosphate buffer saline, PEO N10 = polyethylene oxide N10, PVP: povidone, PVPK-17PF; polyvinylpyrrolidone + 17-PF, VA; vinyl acetate, VA64: polyvinylpyrrolidone, VH: vitreous humor, α-AAA: alpha-amino adipic acid. Dose*; T: treatment, U: untreated, C: control, ↑: increases, ↓: decreases, d: day, h: hour, w: week, m: month, ip: intraperitoneal

**Table 8:** Polymer encapsulated phytochemicals used for in-vitro retinopathy remedy acting against angiogenesis, apoptosis, inflammation, and oxidative stress

Delivery polymer system	Encapsulation	Phytochemical	Stressor	Dose*	Effectiveness	References
Hydrogel (suspension)	Chi + NaA	Lutein (L)	150 µM H_2_O_2_ in ARPE-19 for 2h.	**T1**: 1, 5, 10, 15, 15, 20 + 50 µM LNC, **T2**: 1, 5, 10, 15, 15, 20 + 50 µM micellar L, **C**: LNC without L in stressed cell.	Effective = 10 µM for micellar L + LNC. T1 + T2 ↓ cell death = micellar L (63%) + LNC (79%). T1 + T2 ↓ intracellular ROS by 2-folds for T1 > >T2. T1 + T2 attenuate ΔΨm = micellar L (67%) + LNC (88%) T1 + T2 ↑ cellular L uptake = LNC (17.6 ng/mg + micellar L (9.5 ng/mg). T1 + T2 ↑ L uptake = LNC 1.5-fold (21.6 ng/mg) + micellar L 2-fold (12.1 ng/mg). T1 ↓ ROS from H_2_O_2_ stress	[156]
Hydrogel	Chi (CCS, HCS) + TPP	Resveratrol (RSV)	**S1**: 1 µg/ml LPS in ARPE-19 cells for 24h. **S2**: 15 mW/cm^2^ UV exposure for 30–90 min	**T1**: 50, 100, 250, 500 + 1000 µg/mL HCS, **T2**: 50, 100, 250, 500 + 1000 µg/mL HCS-NG, **T3**: 50, 100, 250, 500 + 1000 µg/mL HCS-RSV-NG, **U**: DR stress, **C**: free RSV without hydrogel in unstressed cell.	**For S1**: HCS, HCS-NG or HCS-RSV-NG at all conc. lacked cytotoxicity on ARPE-19, cell viability at 24h with 100%. No morphological or adhesive changes in ARPE-19 after T. All T conc. did not incite IL-6 + IL-8 inflammation expression compared to untreated DR stress. **For S2**: free RSV photo-degradation = 29% (t = 30 min) + 36% (t = 90 min). HCS-RSV encapsulation mitigated RSV photo-degradation = 15% (t = 30 min) + 17% (t = 90 min). HCS-NG-RSV encapsulation mitigated RSV photo-degradation = 27% (t = 30 min) + 33% (t = 90 min).	[164]
Hydrogel	Chi + TPP	R, Sl + Sg	**S1**: 150 µL DMSO for 4h + 24h in HCE-T, ARPE-19 cells. **S2**: HCE-T, ARPE-19 cells permeability test for 1h	**T1**: 0.062, 0.125, 0.25, 0.5 + 1.0 mg/mL NSL, **T2**: 0.062, 0.125, 0.25, 0.5 + 1.0 mg/mL SL, **T3**: 0.062, 0.125, 0.25, 0.5 + 1.0 mg/mL NSG, **T4**: 0.062, 0.125, 0.25, 0.5 + 1.0 mg/mL SG, **T5**: 0.025, 0.05, 0.1, 0.2 + 0.4 mg/mL for NR, **T6**: 0.025, 0.05, 0.1, 0.2 + 0.4 mg/mL R, **U**: DR stress, **C**: DMSO (-ve) + DMEM (+ ve) in stressed cell.	All T1 to T4 < 1 mg/mL cytotoxicity < 10% for both cell lines, compared to the negative control with > 80% cytotoxicity in 4h + 24h incubation. T5 + T6 permeability coefficient = 3.41 × 10^− 5^ + 3.24 × 10^− 5^ cm/s, respectively in HCE cell line in 1h. T5 + T6 permeability coefficient = 3.39 × 10^− 5^ + 3.60 × 10^− 5^ cm/s, respectively in ARPE-19 cell in 1h	[165]
Liposome	SL (softisan 100, gelucire 44/14), LL (capryol 90) + sur (tween 80)	Dio	20 ng/mL TNF-α in ARPE-19 cells for 48h	**T1**: 0.0025, 0.005, 0.0075, 0.01, 0.025, 0.05, 0.1, 0.25 + 0.5% v/v of NLC, **T2**: 80, 160 µM of Dio-NLC, **C**: 0.1, 1, 10, 50, + 100 µM Dio in unstressed cell.	All Dio conc. reduce cell viability in dose-dependent manner. Only control (100 µM) cause 54% cell death. All T1 = no significant reduction of cell viability. T2 (80 µM)= ↓ cell viability at high conc. (0.25–0.5% v/v). T2 (160 µM) = cytotoxic at conc. 0.025% v/v, but non-cytotoxic at low conc. (0.0025-0.01% v/v). T2 (160 µM) = reversed inflammation at non-cytotoxic conc. (0.005–0.01% v/v)	[166]
Liposome	SL (compritol888), oil (miglyol812, + DMPC) + sur (lutrolF68)	MGN	No stressor in HET-CAM + ORAC	**T1**: 100 µL MGN, **T2**: 100 µL NLC, **T3**: 100 µL MGN-NLC, **C1**: 300 µL of 0.9% NaCl (-ve), **C2**: 300 µL of 0.1 N NaOH (+ ve) in unstressed cell.	Antioxidant activity in ORAC = T1; 3521 µM TE/g, T2; NLC; 769 µM TE/g, T3; 6494 µM TE/g. T3 lack damage to blood vessel of HET-CAM after 5 min of contact. T3 irritation score = < 1, no haemorrhage, lysis, or coagulation, while irritation score of C1 = 0, C2 = 18.82. Percentage haemolysis = T2; 11.16% + T3; 8.10%	[167]
Polymeric particle	BSA + Glut	Curcumin (Cur), rosmarinic acid (R), ursolic acid (UrsA)	30 µg/mL H_2_O_2_ in ARPE cells for 5h	**T**: 0, 5, 10, 30, 50, 100 + 200 µM Cur-BSA, **T2**: 0, 5, 10, 30, 50, 100 + 200 µM R-BSA, **T3**: 0, 5, 10, 30, 50, 100 + 200 µM UrsA-BSA, **C**: PBS in stressed cell.	All T1-T3 = high cell viability > 90% in 18h + 24h incubation. T2 + T3 (50 µM) = better protection from ROS production than cur-BSA. T2 + T3 (30 + 50 µM)= ↑ SOD1 gene expression than cur-BSA. T2 (50 µM)= ↑ GPX1 gene expression than T1 + T3 at 50 µM. T1 + T2 effect = concentration-dependency for inducing GPX1 gene expression.	[168]
Polymeric particle	Chi, NaA + oleic acid core	Lutein (L)	150 µM H_2_O_2_ + 575 µM CoCl_2_ in ARPE-19 for 2h	**T1**: 0, 1, 5, 10, 15 + 20 µM LNC, **T2**: 0, 1, 5, 10, 15 + 20 µM micellar L, **C**: LNC without L in stressed cell.	Effective = 10 µM of T1 + T2. Attenuate ΔΨm = T1 (90%) + T2 (69%) + ↑ T1; 1.4-fold in H_2_O_2_ + 1.5-fold in CoCl_2_, ↑ T2; 1.2-fold in H_2_O_2_ + 1.3-fold in CoCl_2_. ↓ intracellular ROS level = T1 (5-fold) + T2 (2-fold). T1 in H_2_O_2_= ↑ SOD (39%), CAT (52%) + GSH (40%) compared to T2. T1 in CoCl_2_= ↑ SOD (53%), CAT (46%) + GSH (38%), compared to T2. T1 in H_2_O_2_= ↓ MDA + protein carbonyl in both cells. ↓ spliced XBP1 = T1 (1.0-fold) + T2 (0.7-fold) ↓ HIF-1α = T1 (1.1-fold) + T2 (0.8-fold) T1 + T2 ↓ VEGF + ATF4. T1 + T2 ↑ HO-1 + NQO1 protein. T1 + T2 ↑ ZO-1 + Nrf2 activation.	[155]
Polymeric particle	PLA or PLGA	Ferulic acid (FA)	No stressor in BMVEC cells + primary cultures of microvascular pericytes	**T1**: 0.25, 0.5, 1, 2.5, 5.0 mg/mL PLA, **T2**: 0.25, 0.5, 1, 2.5, 5.0 mg/mL PLGA, **T3**: 0.25, 0.5, 1, 2.5, 5.0 mg/mL PLA-FA, **T4**: 0.25, 0.5, 1, 2.5, 5.0 mg/mL PLGA-FA, **C**: DMEM (+ ve) in unstressed cell.	↑ cell viability (> 90%) for T (0.25-1 mg/mL) of T1 + T2 at 24h + 48h in both cells. ↓ cell viability (> 30%) for T (2.5-5 mg/mL) of T1 + T2 at 24h + 48h in both cells. T3 encapsulation efficiency = 75.16%. T4 encapsulation efficiency = 64.86%. FA release in T3 was 50% after 5h, T4 was 50% after 4h and both T3 + T4 was 100% after 24h	[169]
Polymeric particle	PLGA or PLGA–PEG–biotin, PVA: additive for the formulation	Lutein (L)	No stressor in ARPE-19	**T1**: 10, 20 + 50 µg/mL L, **T2**: 10, 20 + 50 µg/mL L-PLGA, **T3**: 10, 20 + 50 µg/mL L-PLGA-PEG-biotin, **C**: DMSO.	Effective = all doses T1-T3. Absence of cytotoxicity in all T1-T3. T2 + T3 ↑ internalization rate for L in the encapsulation, compared to T1 after 6–12h	[170]

Abbreviation: BMVEC: bovine microvascular endothelial cells, BSA: bovine serum albumin, CAT: catalase, Chi: chitosan, CoCl_2_: cobalt(II) chloride, CSS: commercial low molecular weight chitosan, Cur: curcumin, Dio: diosmin, DMPC: 1,2-dimyristoyl-sn-glycero-3-phosphocholine, FA: ferulic acid, Glut: glutaraldehyde, GSH: glutathione, HCS: high molecular weight chitosan, HCS-NG: high molecular weight chitosan nanogel, HET-CAM: hen’s egg test on chorioallantoic membrane, L: lutein, LL: liquid lipid, LNC: lutein loaded nanocarrier system, MDA: malondialdehyde, MGN: mangiferin, NPA: empty poly (lactic acid), NPB: empty poly (lactic-co-glycolic acid), PEG: polyethylene glycol, PLA: poly (lactic acid), PLGA: poly (lactic-co-glycolic acid), PVA: polyvinyl alcohol, R: rosmarinic acid, RSV: resveratrol, Sg: savory, Sl: sage, SL: solid lipid, SOD: super-oxide dismutase, STF: simulated tear fluid, Sur: surfactant, sus: suspension, TPP: tripolyphosphate, UrsA: ursolic acid

Dose*: T: treatment, U: untreated, C: control, ↑: increases, ↓: decreases, d: day, h: hour, min: minute, w: week

## Updates on polymers useful for ocular delivery using 3D printing

The field of 3D printing technology in ophthalmology for ocular drug delivery is new and is based on different tool modalities [171], the material printability [172], and the intended ophthalmological purpose. Examples include personalised lens for optical correction, ocular tissue grafting and engineering for surgical transplantation, other WOD accessories embedded with drugs for treatment [173] or with biosensors for monitoring physiological parameters [174]. In ocular therapy, the ocular implants are used to restore vision, relieve intraocular pressure, or replace damaged eye structures. They often require surgical intervention; an example is the intraocular lens for correcting cataracts. The ocular inserts (OI) are a thin, sterile, multilayered device of different shapes and sizes used to deliver medication directly to different sites of the eye over an extended time. They are typically classified as insoluble, soluble, or bio-erodible based on solubility and often placed under the eyelid or conjunctiva sac without surgical intervention [175]. On the other hand, contact lens can be soft or rigid, curved, transparent and oxygen permeable, placed directly on the cornea to correct refractive errors, provide therapeutic benefit or for cosmetic protection [176].

Different polymer properties make 3D printing technology suitable for non-biocompatible structural models (metallic prosthetic part), biocompatible/non-biodegradable products (lens), biocompatible/biodegradable products (implants and inserts), and biomimetic 3D structures with living cells (hybrid organs). However, for ocular drug delivery purposes, the best choice of polymer for 3D printed WOD should be flexible for personalised fabrication, biocompatible to reduce cytotoxicity to the eye and biodegradable to bypass removals.

The Food and Drug Administration (FDA) has approved WOD fabricated with hydrogels for drug delivery, although not 3D printed, and their application may reduce the need for regular ocular injections of synthetic drugs. Some examples of these implants include Ozurdex^®^, Vitrasert^®^, Iluvien^®^, I-vation^®^, Dextenza^®^ [144] and Mydriasert^®^ [177]. These WOD are easy to administer, can last months or years with sustained release of drugs. In addition, their biodegradability does not require surgical appointments for removal, as the polymers are gradually dissolved by the eye fluid with no side effects [178].

Currently, experimental studies characterizing 3D printed hydrogels are in the spotlight with complex formulations and rheology to improve their usability, including polycaprolactone (PCL) implants embedded with TA. This implant matrix showed a uniform distribution of the drug across the surface, and the cumulative release of TA was within 180 days in-vitro. This PCL implant was biocompatible with over 90% cell viability on ARPE-19 cells [179]. Another study used sodium hyaluronate and sodium alginate hydrogel, with liposome (SL) moxifloxacin (MOX) to 3D print an OI which had 80% encapsulation efficiency. The inserts’ in-vivo drug profile showed 71.2% release in 30 min, slightly greater by 12% than in MOX inserts without liposomal encapsulation. The presence of lecithin liposomes in the mix was suggested to improve MOX solubility and the bio-printing improved the uniformity and stability [180].

Other findings on 80:20% polyethylene glycol diacrylate (PEGDA) and polyethylene glycol 400 (PEG 400) mixed with DEX was used to fabricate a wearable punctual plug. The plug had a sustained release of DEX for 7 days. In comparison, a plug with 100% PEGDA without PEG 400, had prolonged release lasting more than 21 days. This demonstrated that the composition of these polymers also influenced the characteristics of the medtech device [181]. In a 3D printed poly-hydroxyethyl methacrylate (PHEMA) hydrogel loaded with Avastin for contact lens fabrication, the drug release profile was around 60% in 25h, and the lens exhibited significant anti-VEGF effects which made it suitable for DR therapy [182].

A study on ARPE-19 retina cell monolayer, 3D printed on Y79 (photoreceptor cells) mixed with alginate/pluronic F127 had biocompatibility that mimicked and improved retinal and photoreceptor cell density and viability of the printed hybrid retina in 7 days [183]. Another study 3D printed fibrous scaffolding using H_2_O_2_ stressed ARPE-19 cells showed 0.5 µM lutein pretreatment caused 40% reduction in pro-inflammatory cytokines (IL-1β and IL-18) by modulating NLRP3 inflammasomes, reversed the oxidative stress, and maintained phagocytosis and barrier function [184].

These advances in 3D printing technology have enabled the fabrication of highly customizable drug delivery systems, allowing for precise control over dosage, release profiles, and formulation composition tailored to individual patient needs. Unlike conventional topical delivery methods, which often offer limited flexibility in dosing and formulation, 3D printing can incorporate specific phytochemicals into personalized delivery platforms. This approach has the potential to enhance bioavailability, improve patient compliance, and optimize therapeutic outcomes by providing targeted and patient-specific treatments. As a result, 3D printing represents a promising advancement in the field of phytochemical delivery, offering advantages that go beyond those of traditional topical methods.

Hydrogels are well studied as injectable fillings in wound dressing, cosmetics, and for WOD limited to corticosteroids and anti-VEGF for retinopathy treatments [182, 185]. Hydrogels in topical liquid formulation for ocular delivery [185, 186] has been studied but the addition of macular carotenoids can change critical characteristics including its printability. Consequently, hydrogels have great potential for 3D printing that can be modelled using diverse natural and synthetic hydrophilic polymers to generate malleable complex structures, improve biodegradable, non-toxic to cells, increase controlled release for long term use, deliver low dosages with high efficiency, and its responsiveness to environmental factors (pH, temperature, light).

## Computational methods for bioprospecting bioactive compounds against specific retinopathy protein targets

The use of computational methods to accelerate the pipeline of drug development from discovery to approval [187] is based on virtual modelling of ligand-receptor relationships for different diseases. This reduces costs on experimental resources and saves substantial time for fast tracked clinical validation [188]. The application of computational and predictive modelling can shorten the phase of explorative bioprospecting of bioactive compounds and protein targets specific to disease initiation and progression pathways [188–190].

Today, the common computational methods can be used to optimise druggable targets, novel compound discovery and drug repurposing for early retinopathy treatments, including network pharmacology, molecular docking, free energy calculation (i.e. molecular dynamics, thermodynamic integration), pharmacokinetic and bioinformatics [191–193]. Network pharmacology approaches shift the traditional concept of “one drug, one target, and one pathway” to “multi-compounds, genes, and pathways” to better reflect the complex relationships between phytochemicals and proteins holistically. In a study by Zhou et al., network pharmacology investigation of *Siraitia grosvenorii* showed its polyphenol’s ability to treat PDR by interacting with diabetic targets including cGMP-PKG, TNF, IL-17, and AGE-RAGE. In addition, the tumor necrosis factor (TNF), prostaglandin-endoperoxide synthase 2 **(**PTGS2), and caspase 3 (CASP3) were suggested to be the core targets for retinopathy treatment from PDR differentially expressed gene sets (GSE60436) by network pharmacology [194]. Also, phytochemicals from Asian herbal medicines like *Astragalus membranaceus* [195], *Lingqihuangban* [196], *Mingmu dihuang* [197], *Panax notoginseng* [198], *Qidengmingmu* [199], *Radix salviae* [200], *Tangwang mingmu* [201], *Xueshuantong* [202], and many other plants [203] have been linked to target retinopathy-specific proteins via network constriction, protein to protein interactions (PPI), enrichment analysis, and co-expression. The outcomes from this approach have been validated and verified in in-vitro, and in-vivo models [61, 190, 204–206] and is therefore useful for selecting potential diagnostic markers and phytochemical specific protein targets specifically oxidative stress, inflammation, apoptosis and angiogenesis (Table 9) evolving in the field of ocular pharmacology.

**Table 9:** Computational methods used to match protein targets with phytochemicals for early retinopathy treatments. Phytochemicals evaluated with more than one computational method are represented in orange

Computational methods	Analysis	Parameters for analysis	Dataset/sequence/protein targets	Protein target link to retinopathy-specific pathway	Pathway mechanism classification	Phytochemicals/drug	Computational outcome	Application	References
Dynamic simulation	RMSD, RMSF + H-bond strength	Simulation timeframe = 100 ns	ALR (pdb id: 1US0) human protein template	Polyol pathway	Hyperglycaemia	Apigenin, ellagic acid, jacquilenin, kaempferol, magnolialide + pelargonidin. Reference drug = tolrestat	**RMSD** of phytochemical-ALR complex range between 0.114 to 0.133 nm, compared to reference drug tolrestat-ALR with 0.146 nm. **RMSF** profile of amino acids residue in phytochemical-ALR complexes has low fluctuation range between 0.1–0.3 nm, which is like the reference drug tolrestat-ALR complex. **H bonds** between phytochemical-ALR complex are between 52–97% compared to tolrestat-ALR complex with 33%. Phytochemicals have reduced mobility and formation of stable complex	Drug discovery	[9]
	RMSD, RMSF, Rg + SASA	Solvation to mimic biological system: TIP3P water model via the ‘gmx solvate’ command. Charges neutralised by adding 150 mM NaCl. NVT + NPT at 300 K, 1.0 bar pressure. Apigenin + isozeylanone topology by CgenFF version of CHARMM-GU. Simulation timeframe = 200 ns	SRC (pdb id: 2BDJ) + HSP90AA1 (pdb: 4BQG)	Rap1 signaling, PI3K–AKT signaling pathway	Differentiation, proliferation, inflammation	Apigenin + isozeylanone	**RMSD** of uncomplex SRC + uncomplex HSP90AA1 ranges between 0.149 to 0.183 nm, while apigenin-SRC complex + isozeylanone-HSP90AA1 complex ranges between = 0.073 to 0.122 nm. **RMSF** profile of amino acids residue in apigenin-SRC complex + isozeylanone-HSP90AA1 complex range between 0.05 to 0.4 nm, and this was similar for uncomplex SRC + uncomplex HSP90AA1. **Rg** of uncomplex SRC + uncomplex HSP90AA1 ranges between 1.716 to 1.903 nm, while apigenin-SRC complex + isozeylanone-HSP90AA1 complex ranges between 1.728 to 1.915 nm. **SASA** of uncomplex SRC = 133.6 nm^2^, apigenin-SRC complex = 134.5 nm^2^, uncomplex HSP90AA1 = 110.02 nm^2^, isozeylanone-HSP90AA1 complex = 111.8 nm^2^. Phytochemical could stabilize the protein complex function.	Drug discovery	[193]
	RMSD, RMSF, Rg + total energy	NVT (500 ps) + NPT (1000 ps) equilibrations. Andrographolide topology by PRODRG2 server. Nrf2-Keap1 topology by GROMOS96 43a1 force field. Simulation timeframe = 20 ns	Nrf2-keap1 (pdb id: 4L7B) human protein template	Nrf2 signalling pathway	Molecular switch in oxidative stress	Andrographolide	**RMSD** confirms andrographolide-Nrf2-Keap1 complex was stable with range between 0.2 to 0.25 nm, and this was at equilibrium from 9–20 ns simulation time. **RMSF** profile of amino acids residues in andrographolide-Nrf2-Keap1 complex has low fluctuation range between 0.1 to 0.5 nm. **Rg** of andrographolide-Nrf2-Keap1 complex range between 0.35 to 0.40 nm. Total energy stabilizes for andrographolide-Nrf2-Keap1 at -525,000 KJ/mol. Phytochemical was stable with minimal conformational changes in protein pocket	Drug discovery	[207]
	RMSD, RMSF, Rg + total energy	Charge neutralizes by sodium ions or chloride ions. Cubic box + water models. NVT (500 ps) + NPT (1000 ps) equilibrations. Tert-butylhydroquinone topology by PRODRG2 server. NFE2L2, MAPK8 + PTGS2 topology by GROMACS. Simulation timeframe = 30 ns	NFE2L2 (pdb id: 4L7B), MAPK8 (pdb id: 4L7F) + PTGS2 (pdb id: 5KIR) human protein template	HIF-1 signalling, VEGF signalling, FOXO signalling, PI3K/AKT signalling, Rap1 signaling pathways, renin-angiotensin system.	Inflammation, oxidative stress, vascular system development, cell proliferation-death regulation	TBHQ	**RMSD** confirms uncomplex TBHQ was stable and ranges between 0.0 to 0.15 nm, also uncomplex NFE2L2, uncomplex MAPK8 + uncomplex PTGS2 ranges between 0.35 to 0.8 nm, and this was at equilibrium from 16–30 ns simulation time. **RMSF** profile of amino acids residues in the uncomplex NFE2L2, uncomplex MAPK8, and uncomplex PTGS2 has low fluctuation range between 0.05 to 0.6 nm, with total energy of − 1,061,000, −1,197,000, and − 526,000 kJ/mol, respectively. **Rg** of uncomplex TBHQ was at 0.24 nm, also uncomplex NFE2L2, uncomplex MAPK8 + uncomplex PTGS2 ranges between 0.25 to 0.36 nm. Phytochemical has stable structural stability for interaction with the proteins	Drug discovery	[208]
	RMSD, RMSF, Rg + SASA	An explicit solvent-simple point charge model (SPC216 water molecules). NVT + NPT for 100 ps at 300 K, 1.0 bar pressure. Albumin topology by amber99sb-ildn force field. Curcumin topology by general amber force field. Simulation timeframe = 50 ns	ALB (pdb id: 1AO6) human protein template	AGE-RAGE signalling, PI3K-Akt signaling pathway	Apoptosis, inflammation, insulin transduction signals	Curcumin	**RMSD** of curcumin-ALB complex remain at equilibrium during the simulation time between 0.3 to 0.4 nm. **RMSF** profile of amino acids residue in phytochemical-ALB complex range between 0.1 to 0.5 nm. **Rg** of curcumin-ALB complex ranges between 3.5 to 3.8 nm. **SASA** of curcumin-ALB complex ranges between 540 to 580 nm^2^ and this show strong density and stability. H bond contributed to the stability. Phytochemical has stable structural stability for interaction with the protein	Drug discovery	[209]
	RMSD + Rg	Simulation timeframe = 10 ns. Parameters not stated	VEGFA (pdb not given)	VEGF signalling pathway	Angiogenesis	Bassic acid, eriodyctiol, epicatechin, ginkgolide A, scutellarin + tetrandrine	Only uncomplex scutellarin RMSD + Rg was known from simulation. **RMSD** of uncomplex scutellarin was between 0.1 to 0.2 nm. **Rg** of uncomplex scutellarin occurs within 2 nm range. The conformational remain stable before and after simulation but with limited bond interaction.	Drug discovery	[210]
	RMSD + RMSF	Simulation timeframe = 100 ns. Parameters not given	ALR2 (pdb id: 4JIH) human protein template	Polyol pathway	Hyperglycaemia	Agnuside, eupalitin-3-O-galactoside, picroside II + 7-O-methylwogonin	**RMSD** of phytochemical-ALR2 complex range between 0.14 to 0.16 nm compared to reference drug epalrestat-ALR2 complex 0.19 nm. **RMSF** profile of amino acids residue in phytochemical-ALR2 complex range between 0.07 to 0.09 nm compared to reference drug epalrestat-ALR2 complex with 0.090 nm. Overall, agnuside-ALR2 was best with high stability among the complexes	Drug discovery	[206]
	RMSD, RMSF, Rg, SASA + PSA	Solvation to mimic biological system: TIP3P water model with periodic boundary condition. Charges neutralised by adding 0.01 M NaCl. NPT with constant temperature of 300 K. Simulation timeframe = 100 ns	DPP4 (pdb id: 6B1E) human protein template	AGE-RAGE signalling, MAPK signalling, chemokine signalling, insulin signalling, HIF-1 signalling, Ras signalling, PI3K/AKT signalling pathway	Inflammation, proliferation, differentiation, metabolism, cytoskeletal reorganisation.	Terchebin, locoracemoside B + TGBG. Reference drug = vildagliptin	**RMSD** of uncomplex DPP4 + terchebin = 0.31 + 0.35 nm, uncomplex DPP4 + locoracemoside B = 0.23 + 0.22 nm, uncomplex DPP4 + TGBG = 0.19 + 0.22 nm compared to reference drug vildagliptin + DPP4 = 0.22 + 0.20 nm. **RMSF** profile of amino acids residues of the phytochemical-DPP4 complex exhibit minimal fluctuation below 0.45 nm. **SASA** of terchebin-DPP4 was most stable among the phytochemicals. **PSA** of oxygen + nitrogen atoms remained stable for terchebin + locoracemoside B but fluctuate for TGBG. Phytochemicals have stable interactions in the DPP4 active site	Drug discovery	[211]
Network pharmacology	Compound-target network, GO + KEGG enrichment	DEG parameter: unknown Enrichment parameter: unknown. PPI parameter: unknown	Processed metabolomic sample from mice	mTOR signaling, AMPK signaling, neuroinflammation signaling, PPAR-α/RXR-α activation, apelin adipocyte signaling, T2DM signaling, NFκB signaling, RAR activation.	Neuroinflammation, Hyperglycaemia, cell survival, proliferation, vascular remodelling	AS-IV, emodin, rhein, chrysophanol, chrysophanol-8-O-β-D-glucopyranoside, aloe-emodin, aloin, biochanin A, 10-hydroxyoleuropein, 3,4′,7trihydroxyflavone, + 3′ME7G.	Core protein targets for retinopathy treatment = ERK1/2, PI3K, p70 S6K, MAPK signal transduction, mTORC1 signaling, + NF-κB signaling pathways	Potential diagnostic biomarker + phytochemical multi-target discovery	[8]
	Disease-target network, PPI, GO + KEGG enrichment	DEG parameter: unknown Enrichment parameter: *Homo sapiens* + FDR cut-off: 0.05. PPI parameter: highest confidence scores > 0.9	Not given	Autophagy, AGE-RAGE signalling pathway.	Inflammation, neurodegeneration, vascular proliferation	None	One hundred and fifteen genes common among retinopathy, autophagy + neurodegeneration. Core functional protein targets = Beclin 1, LC3, NFκB, TNF, IL1 + IL6	Potential diagnostic biomarker	[61]
	Compound/disease-target network, PPI, GO + KEGG enrichment	DEG parameters: logFC > 1 or − 1, + p-value = 0.05. Enrichment parameters: Log2FC. Version/species/type; “Ensemble 104 or 51”, “*Homo sapiens* (GRCh38. p13)” + p value ˂ 0.05. PPI parameters: Species-*Homo sapiens*, confidence cutoff; 0.4	GSE60436 (GEO human dataset)	Fluid shear stress, atherosclerosis, chemical carcinogenesis-DNA adducts, IL-17 signalling, AGE-RAGE signalling pathway.	Apoptosis, angiogenesis, inflammation, oxidative stress, arachidonic acid metabolism	Beta-sitosterol, flazin, kaempferol, mandenol, perlolyrine + ZINC03860434	Eighty-five protein targets discovery for all phytochemicals. 18 proteins intersect between phytochemical + retinopathy dataset. 7 downregulated expressions = CHRM3, KCNH2, ACHE, GSTM1, GSTM2 + CYP1B1. 11 unregulated expressions = PIK3CG, CASP3, STAT1, TNF, NOS3, HMOX1, CDK1, PTGS1, ADRA1B, MMP1 + PTGS2. Top core protein targets for the phytochemicals = TNF, CASP3 + PTGS2	Phytochemical multi-target discovery	[194]
	Compound/disease-target network, PPI, GO + KEGG enrichment	DEG parameter: FPKM > 5, log 2FC ≥ 1, + P-value ≤ 0:05 Enrichment parameter unknown. PPI parameter: unknown	Total RNA of HRMEC	Nrf2 signaling pathway	Apoptosis, autophagy, inflammation, cell cycle, vascular system development, molecular switch in oxidative stress	Andrographolide	Andrographolide modulated the Nrf2 signaling pathway, balanced glutathione metabolism, regulated arachidonic acid metabolism and affect mitochondrial oxidative phosphorylation to resist HRMECs oxidative stress injury. Andrographolide influence expression of NOS3, SNAI1 + F-actin to promotes cell migration, regulate endothelial barrier, affect cell adhesion + inhibit cytoskeleton damage. Core gene are NFE2L2 + HMOX1, and Nrf2	Phytochemical multi-target discovery	[207]
	Compound/disease-target network, PPI, GO + KEGG enrichment	DEG parameter: unknown Enrichment parameter: *Homo sapiens* + FDR cut-off: 0.05. PPI parameter: high confidence scores > 0.7	Not given	HIF-1 signalling, VEGF signalling, FOXO signalling, PI3K/AKT signalling, Rap1 signaling pathways, renin-angiotensin system.	Inflammation, oxidative stress, vascular system development, cell proliferation-death regulation	TBHQ	Eighty-five protein targets intersect between phytochemicals and retinopathy dataset. Top core protein = MAPK8, SRC, RELA, ESR1, APP, NOS3, MAPK14, ALB, PTGS2 + AHR.	Phytochemical multi-target discovery	[208]
	Disease-target network, GO, KEGG enrichment + gene-disease association	DEG parameter: unknown Enrichment parameter: unknown. PPI parameter: unknown	GSE3249 (GEO mice dataset) + DAPK1 (pdb id: 2YAK)	Sphingolipid signalling, AMPK signalling, PI3K-Akt signalling, insulin signalling, C-type lectin receptor, Hippo signalling, TNF signalling, IL-17 signalling, Ras signalling, p53 signalling, Rap1 signalling, VEGF signaling pathway.	Apoptosis, inflammation, neurodegeneration,	None	Highly expressed protein = EEF2, HSP90AA1, PKM, TUBB4B, ACTC1, TPM3, YWHAB, UBC, YWHAQ, CAPZB, CALM1 + CALM2. DAPK1 associated with 4 eye disease categories = eye tumour, retinal degeneration, corneal disease + optic-related disorders	Potential diagnostic biomarker	[212]
	Compound/disease-target network, PPI, GO + KEGG enrichment	DEG parameter: Unknown. Enrichment parameter: unknown. PPI parameter: minimum confidence score of 0.400. Similarity cut-off of 0.85	Not given	PI3K-Akt signalling, MAPK signalling pathway, Ras signalling, chemokine signalling, insulin signalling, HIF-1 signalling, AGE-RAGE signalling pathway	Inflammation, proliferation, differentiation, metabolism, and cytoskeletal reorganisation.	Camphor, cinnamic acid, curcumin, chrysin, kaempferol 3-O-beta-D-galactoside, strychnine, kaempferol, oleanolic acid, palmitic acid, ellagic acid, oleic acid, quercetin, quercitrin, stearic acid, caffeic acid, sucrose, ursolic acid, + ascorbic acid.	Thirty-seven protein targets were common among diabetes complications. Proteins involved in multiple pathways = AKT1, AKT2, PIK3Cγ, PIK3Cα, PTEN. Hub proteins with maximal interaction = carbonic anhydrases like CA2, CA7, CA4, CA12 and CA12 + EGFR, AKR1B1, MMPs, Aryl hydrocarbon receptor. Key obesity-associated transcription factors = PPARα, PPARβ. Pro-inflammatory mediators = IL1α, IL10, TNFα, CCL2, TGFβ1, NFKβ1.	Phytochemical multi-target discovery	[211]
	Disease-target network, PPI, GO, KEGG enrichment + ROC curve monofactor analysis	DEG parameters: log2 fold-change (FC)| ≥ 2 + FDR < 0.05. Area under the ROC curve > 0.9 = great diagnostic value. Enrichment parameter: unknown. PPI parameter: unknown	GSE60436 + GSE102485 (GEO human dataset)	Intrinsic apoptotic signaling in response to DNA damage, ROS metabolic process, response to oxidative stress, p53 signaling	Apoptosis, inflammation, oxidation	None	Top core protein = TXNIP, CD44, HMOX1, NCF2, ALOX5, TLR4, PTGS2, TP53, NOX4 + CAV1. Diagnostic accuracies of CAV1, CD44, NOX4, TLR4, and TP53 for retinopathy were 96.97%, 100.00%, 96.97%, 96.97%, + 98.48% in GSE102485 dataset, respectively.	Potential diagnostic biomarkers	[213]
	Compound/disease-target network, PPI, GO + KEGG enrichment	DEG parameter: unknown. Enrichment parameters: p-value < 0.05 + Q-value < 0.05. PPI parameter: Unknown	Not given	PPAR signaling, VEGF signalling,	Angiogenesis, hyperglycaemia, inflammation, neovascularization	Beta-carotene, sophoranol, oleanic acid + stigmasterol.	63 proteins intersect between phytochemicals + retinopathy. Top core protein targets = MAPK3, RELA, ESR1, PRKCA, BCL2, HIF-1α, PTGS2, IL6 + VEGFA.	Phytochemical multi-target discovery	[214]
	Disease-target network, GO + KEGG enrichment	DEG parameters: log2 fold-change (FC)| ≥ 2 + FDR < 0.05. Enrichment parameter: unknown.	GSE11733, GSE19122, GSE28831 + GSE55389 (GEO mice and rat dataset)	Cytokine signaling in immune system, interferon α/β signaling, TGFβ1 signaling, RAGE activation,	Apoptosis, angiogenesis, DNA fragmentation	None	The common pathways enriched in GSE11733 = amino acids metabolism, sodium-coupled sulphate, di- and tri-carboxylate transporters (Nact), activation of NEK6, NEK9, + NrCAM interactions. All pathways are inter-connected with the core proteins = SLC13, CFLAR, SCTR, FSHB, RLN3, ADR1D, ADR2C, CHRM5, LPAR2, FSHR, THRB, GLRA3, PRSS3, KCNJ16, KCNE2, ATP4A, ATP1B4, ADCY7 + PTGES.	Potential diagnostic biomarkers	[215]
	Disease-target network, GO + KEGG enrichment	DEG parameter: p-value < 0.05 + log FC > = 0.5–1.5. Enrichment parameter: FDR p-value < 0.05. PPI parameter: high confidence scores > 0.7	GSE140959 GSE57362 GSE60436 (GEO human dataset)	AGE-RAGE signaling, chemokine signaling, TNF signaling, toll-like receptor signaling, NOD-like receptor signaling, PI3K-Akt signaling pathway	Angiogenesis, inflammation, neurogenesis, blood vessel development, extracellular matrix organization	None	9 differential expressed genes + methylation pattern in the mild NPDR. 5 upregulated gene = NR1H4, ROCK2, HTATIP2, UHRF1 + NTM. 4 downregulated gene = MAPT, FAM69C, FHOD3 + IGSF21. Top 7 core gene with > 5 interaction = FN1, IL6, COL1A2, COL4A1, COL4A2, SPARC + MMP9.	Potential diagnostic biomarkers	[216]
	Disease-target network, PPI, GO, KEGG enrichment + co-expression	DEG parameter: unknown Enrichment parameter: *Homo sapiens* + FDR cut-off: 0.05. PPI parameter: highest confidence scores > 0.9. Co-expression parameter: p-value ≤ 0.005	GSE146615 (GEO human dataset)	Il-1/12 signalling, wnt signalling cytokine mediated signalling, VEGF signalling	Apoptosis, angiogenesis, inflammation, oxidation	None	88 proteins intersect between autophagy + retinopathy disease. Top protein of autophagy = APP, ATG7, GNAI3, HDAC1, HSP90AA1, HSPA8, KRAS, PIK3R1, TP53, UBB + UBR4. Core co-expressed proteins between autophagy + retinopathy = TP53, HSAP90AA1, APP + PIK3R1	Potential diagnostic biomarker	[217]
	Compound/disease-target network, PPI, GO + KEGG enrichment	DEG parameter: unknown. Enrichment parameter: FDR cut-off: 0.05. PPI parameter: minimum confidence scores > 0.4.	Not given	AGE-RAGE signalling, IL-17 signalling, fluid shear stress	Apoptosis, inflammation, oxidation	Huperzine A	20 proteins intersect between huperzine A + retinopathy. Top core protein for retinopathy = HSPB1 (HSP27), Bax, Bcl­2 + Caspase3 Core protein enriched pathways = AGE-RAGE signalling pathway in diabetic complications, apoptosis pathway, IL-17 signalling pathway + fluid shear stress and atherosclerosis.	Phytochemical multi-target discovery	[218]
Molecular docking	Binding affinity	Not given	AKT1 (pdb id: 1UNQ), HIF1A (pdb id: 4H6J), TNFα (pdb id: 5YOY) and VEGFA (pdb id: 4ZFF) human protein template	VEGFA signaling, HIF-1 signaling, PI3K-Akt signaling, NFκB signaling, apoptosis-related signaling pathway	Apoptosis, angiogenesis, hypoxia, inflammation	Baicalein, luteolin, kaempferol + quercetin	The binding affinity range= -5.5 to -6.4 kcal/mol. Hydrogen bonds contributed to the affinity. Baicalein-HIF1A (-6.4 kcal/mol) has best affinity. Kaempferol-TNFα (-5.5 kcal/mol) has least affinity	Phytochemical intramolecular strength	[199]
	Binding affinity + bond interaction	AKT1 grid center = x; 32, y; 42, + z; 13. Grid box size x; 64, y; 64 + z; 64Å. SRC grid center = x; −3.1, y; 51 + z; 25. Grid box size x; 90, y; 77 + z; 104Å. VEGFA grid center = x; −39, y; −57, + z; −3. Grid d box size x; 65, y; 138 + z; −365Å	AKT1 (pdb id: 4EJN), SRC (pdb id: 4U5J), + VEGFA (pdb id: 3BDY) human protein template	AGE-RAGE signaling, PI3K-AKT signaling, Rap1 signaling, HIF-1 signaling pathway”	Apoptosis, angiogenesis, oxidation, inflammation, neovascularization, hyperglycaemia, hypoxia	Acacetin, alisol B, luteolin + naringenin	The binding affinity range= -6.9 to -9.7 kcal/mol. Luteolin-AKT1 (-9.7 kcal/mol) has best affinity. Naringenin-VEGFA (-6.9 kcal/mol) has least affinity. Luteolin bind AKT1 amino acids residue at Ser205, Asp292, Gln79 + Asn54. Other amino acids residue contributing to the affinity = Tyr340, Met341, Leu273 + Thr338 in SRC. Ser168, Asp167, Gln107, Gly41, Gln38 + Gly42 in VEGFA	Phytochemical intramolecular strength	[197]
	Binding affinity + bond interaction	Grid box = x; 60 × y; 60 × z; 60 at a grid resolution of 0.375 Å	VEGF (ID not given)	VEGF signalling pathway	Angiogenesis	Bassic acid, eriodyctiol, epicatechin, ginkgolide A, scutellarin + tetrandrine. Reference drug = triamcinolone	Phytochemicals binding affinity range= -5.05 to -6.02 kcal/mol. Hydrogen + van deer waal bonds contributed to the affinity. Tetrandrine-VEGF (-6.02 kcal/mol) has best affinity. Ginkgolide A-VEGF (-5.05 kcal/mol) has least affinity. Reference drug affinity= -5.18 kcal/ mol. Tetrandrine bind VEGF amino acid residue at Val7, Arg16, Lys9, Asp12 + Ile69. Amino acids residue contributing to affinity by hydrogen bond = Thr70, Gln72, Glu68, Asn68 + Arg16. Amino acids residue contributing to affinity by van deer waal bond = Lys9, Asn68, Val8 Lys9, Asp12, Ile69, Arg16, Glu23, Gln91 + Leu47	Phytochemical intramolecular strength	[210]
	Binding affinity + bond interaction	Active site residues of DPP4; Glu205, Glu206, Asp708, His740, Ser630, Arg125, Ser209, Phe357, Tyr547, Tyr631, Ile651, Trp659, Tyr662, Tyr666, Arg669 + Val711 define the grid box.	DPP4 (pdb id: 6B1E) human protein template	PI3K-Akt signalling, MAPK signalling pathway, Ras signalling, chemokine signalling, insulin signalling, HIF-1 signalling, AGE-RAGE signalling pathway	Inflammation, proliferation, differentiation, metabolism, cytoskeletal reorganisation	1,2,4,6 tetra o galloyl beta d glucose, tercatain, 1,6-bis-o-galloyl-beta-d-glucose, glucogalin, epicatechin, 1,6-bis-o-galloyl-beta-d-glucose kotalanol, benzoylsalireposide, procyanidin, salirepin, myricitrin, bisacurone, catechin, sucrose, chebulinic acid, kaempferol-3-o-beta-d-galactoside, proanthocyanidin, kaempferol-7-o-a-rhamnoside, quercitrin, kaempferol-7-oglucoside, punicafolin, locoracemoside b, narcissin, corilagin, quercetin, rutin, typhaneoside, salacinol, symplocomoside, luteolin, symplososide, symploveroside, epicatechin-4b-8, symponoside, terchebin. Reference drug = vildagliptin	Phytochemicals binding affinity range= -5.317 to -11.766 kcal/mol. Hydrogen, hydrophobic, ionic bond + water bridge contributed to the affinity. Terchebin-DPP4 (-11.766 kcal/mol) has best affinity. Symponoside-DPP4 (-5.317 kcal/mol) has least affinity. Reference drug = vildagliptin-DPP4 -3.899 kcal/mol. Terchebin bind DPP4 amino acid residue with hydrogen bond = Glu206, Ser242, Tyr545 Arg560 + Asp739. Terchebin bind DPP4 at amino acid residue with hydrophobic bond = Lys122, Arg125, Tyr547, Trp627 + Trp629.	Phytochemical intramolecular strength	[211]
	Binding affinity + bond interaction	Box side length = 30 Å	PKC-α, ERK1/2, VEGFA + HIF-1α (ID not given)	PPAR signaling, VEGF signalling,	Angiogenesis, hyperglycaemia, inflammation, neovascularization	Oleanolic acid	Phytochemical binding affinity range= -5.4 to -9.2 kcal/mol. Hydrogen, hydrophobic bond + pi-pi stack contributed to the affinity. Oleanolic acid-PKC-α (-9.2 kcal/mol) has the best affinity. Oleanolic acid-HIF-1α (-5.4 kcal/mol) + Oleanolic acid-ERK2 (-5.4 kcal/mol) has the least affinity. Amino acids residue contributing to affinity by hydrogen bond = Glu225, Asp34, Lys409, Ser151 + Asp184. Amino acids residue contributing to affinity by hydrophobic bond = Met319, Gly59, Gly58, Asp475, Ala521, Lys149, Gly51 + Glu50. Amino acids residue contributing to affinity by pi-pi stack = Trp190 + Tyr111	Phytochemical intramolecular strength	[214]
	Binding affinity + bond interaction	Grid box binding centre = x; 19.8179, y; 24.2625 and z; 62.6601	ALR (pdb id: (2R24) human protein template	Polyol pathway	Hyperglycaemia	Mangiferin	Phytochemical binding affinity magniferin-ALR for pose 1= -34.37 kcal/mol. The binding affinity for magniferin-ALR pose 2= -35.46 kcal/mol. Hydrogen bonds, van der waal bond, pi sigma + pi-pi bond contributed to affinity. Mangiferin bind ALR at amino acids residue with hydrogen bond = Lys76, Gln182, Typ47, Leu299 + Trp110. Mangiferin bind ALR at amino acids residue with van der waal = His109, Ser158, Ile259, Asp42, Ser209, Ser213, Trp19, Val296, Arg216, Ala298 + Leu300. Mangiferin bind ALR at amino acids residue with pi sigma bond = Ala297. Mangiferin bind ALR at amino acids residue with pi-pi stacking = Typ208	Phytochemical intramolecular strength	[205]
	Binding affinity	Not given	VEGF (ID not given)	VEGF signalling pathway	Apoptosis, angiogenesis, oxidation	Myricetin	9 fitting mode of myricetin-VEGF binding affinity range= -3.2 kcal/mol to -9.3 kcal/mol. The first fitting mode of -9.3 kcal/mol has best affinity.	Phytochemical intramolecular strength	[219]
	Binding affinity + bond interaction	Hydrogen, kollman charges + gasteiger charges added to complex. LGA = 2,500,000 energy, maximum number of generations = 27,000	PKCα (pdb: Id 1A25) human protein template	DAG– PKC signalling pathway	Hyperglycaemia, oxidation	Maleimide derivative 3, bisindolylmaleimide I, CHEMBL311543, CHEMBL316239. Reference drug = ruboxistaurin	Phytochemical binding affinity range= -8.88 to -9.36 kcal/mol. Hydrogen bonds contribute to the affinity. Maleimide derivative 3-PKCα (-9.36 kcal/mol) has the best affinity. CHEMBL311543- PKCα (-8.88 kcal/mol) has the least affinity. Reference drug affinity= -8.61 kcal/mol. Maleimide derivative 3 binds PKCα at amino acids residue with hydrogen bond = Arg159, Gly257 + Phe255. CHEMBL311543 binds PKCα at amino acids residue with hydrogen bond = Arg159	Phytochemical intramolecular strength	[220]
	Binding affinity	Not given	RAGE (ID not given)	AGE-RAGE signalling pathway	Inflammation, oxidation	Curcumin, lutein + zeaxanthin. Reference drug = RAGE antagonistic peptide	Phytochemical binding affinity range= -7.9 to -10.0 kcal/mol. Lutein-RAGE (-10.0 kcal/mol) has the best affinity. Curcumin-RAGE (-7.9 kcal/mol) has the least affinity. RAGE antagonistic peptide binding affinity= -5.6 kcal/mol	Phytochemical intramolecular strength	[221]
	Binding affinity	Not given	ANG2 (pdb id: 1Z3S) human protein template	Tyrosine kinase receptor signalling pathway	Angiogenesis, hypoxia, neovascularization	D-pinitol, allicin, ajoene, salacinol, tolazamide, nateglinide, tolbutamide + biguanide	Phytochemical binding affinity range= -1.38 to -5.05 kcal/mol. Tolazamide-ANG2 (-5.05 kcal/mol) has the best affinity. Salacinol-ANG2 (-1.38 kcal/mol) has the least affinity. Hydrogen bond + hydrophobic bond contributed to the affinity.	Phytochemical intramolecular strength	[222]
	Binding affinity	Grid box based on protein active sites	AKT1, VEGF-A, IL-6, TNF, NOS3, PPARG, IL-10 + MMP9 (ID not given)	AGE-RAGE signalling, MAPK signaling, PI3K-AKT signaling, HIF-1 signaling, TNF signaling, IL-17 signalling pathway	Angiogenesis, apoptosis, inflammation, oxidation, hypoxia, neovascularization	Isorhamnetin, kaempferol, luteolin, quercetin	Phytochemicals binding affinity range= -6.6 to -10.8 kcal/mol. Luteolin-MMP9 (-10.8 kcal/mol) has the best affinity. Kaempferol-IL6 and Kampferol-IL10 (-6.6 kcal/mol) both has the least affinity.	Phytochemical intramolecular strength	[223]
Pharmacokinetics	Absorption, distribution, excretion + toxicity	Not given	Not applicable	Polyol pathway	Hyperglycaemia	Apigenin, Hexadecane, heptadecanone, pelargonidin, magnolialide, kaempferol, intybulide A + jacquilenin	All phytochemical solubility range = 0.0760085 to 14,652.2 mg/L. BBB penetration score = 0.287 to 25.34. Caco-2 cell permeability range = 1.29 to 22.26 nm/s. MDCK cell permeability range = 1.519 to 68.012 nm/s. Skin permeability (Log Kp) range= -3.05 to -0.550 cm/h. PPB efficiency range 60–100%. HIA = 75 to 100% LD_50_ range = 750–5000 mg/kg. No mutagenicity, carcinogenicity + cytotoxicity	Phytochemical safety	[9]
	Absorption, distribution + excretion	Not given	Not applicable	VEGF signalling pathway	Angiogenesis	Bassic acid, eriodyctiol, epicatechin, ginkgolide A, scutellarin + tetrandrine	All phytochemical solubility range= -2.2 to -8.02 mg/mL. High gastrointestinal absorption, except scutellarin. Lack BBB penetration ability. Lipophilicity range = 1.11 to 5.16. All phytochemicals are P-gp substrate except tetrandrine.	Phytochemical safety	[210]
	Absorption, distribution, metabolism, excretion + toxicity	Not given	Not applicable	Polyol pathway	Hyperglycaemia	Magniferin	Mangiferin is soluble in water (1.054 mg/mL). Low transport across gastrointestinal barrier. Lack blood barrier permeation. Poor oral bioavailability. Renal clearance = 1.119 mL/min. Half-life = 1.162h. HIA = 58.71%. Not P-gp substrate. hERG affinity pKi = 3.38. hERG activity pIC50 = 3.08. Lack ability to inhibit CYP1A2, CYP2C19, CUP2C9, CUP2C9, CYP2D6 + CYP3A4	Phytochemical safety	[205]
	Absorption, distribution, metabolism, excretion + toxicity	Not given	Not applicable	NFκB signalling pathway	Apoptosis, inflammation	Caulerpin, caulersin racemosin A, racemosin B + racemosin C	All phytochemicals are P-gp substrate. Volume of distribution range= -0.881–0.365. High gastrointestinal absorption = 95.097 to 100. LD_50_ range = 2.336–2.661 mol/kg. Caco-2 cell permeability range = 1.29–22.26 nm/s. Total clearance range = 0.320–1.249 mL/min. Phytochemicals are non-inhibitor of hERG I BBB permeability range= -0.696 to 0.232. All phytochemicals can inhibit CYP3A4 + CYP2C19. Phytochemicals are substrate for CYP3A4, but not CYP2D6. Phytochemicals are hepatotoxic except racemosin A	Phytochemical safety	[224]

Abbreviation: 3′ME7G: 3′-O-methyl-(−)-epicatechin 7-Oglucuronide, ACTC1: actin alpha cardiac muscle 1, ADCY7: adenylate cyclase 7, ADRA1D: adrenoceptor alpha 1d, ADRA2C: adrenoceptor alpha 2c, AGE-RAGE: advanced glycation end-products and its receptor, AHR: aryl hydrocarbon receptor, AKT: serine/threonine kinase, ALB: albumin, ALI: aqueous solubility, ALR2: aldose reductase 2, AMPK: adenosine monophosphate activated protein kinase, APP: amyloid beta precursor protein, APP: amyloid beta precursor protein, AS-IV: astragaloside IV, ATG7: autophagy related 7, ATP1B4: atpase Na+/K + transporting subunit beta 4, ATP4A: atpase H+/K + transporting subunit 4 alpha, BBB: blood brain barrier, Caco-2: human epithelial colorectal adenocarcinoma cell line, CALM1: calmodulin 1, CALM2: calmodulin 2, CAPZB: capping actin protein of muscle z-line beta subunit, CFLAR: casp8 and fadd like apoptosis regulator, CgenFF: charmm general force field, CHRM5: cholinergic receptor muscarinic 5, COL1A2: collagen type i alpha 2 chain, COL4A1: collagen type iv alpha 1 chain, COL4A2: collagen type iv alpha 2 chain, CYP1A2: cytochrome p450 1a2, CYP2C19: cytochrome p450 2c19, CYP2C9: cytochrome p450 2c9, CYP2D6: cytochrome p450 2d6, CYP3A4: cytochrome p450 3a4, DAG-PKC: diacylglycerol-protein kinase c, EEF2: eukaryotic translation elongation factor 2, ESOL: estimated solubility, ESR1: oestrogen receptor 1, FDR: false discovery rate, FOXO: forkhead box O, FN1: fibronectin 1, FRKM: fragments per kilobase of exon per million mapped reads, FSHB: follicle stimulating hormone subunit beta, FSHR: follicle stimulating hormone receptor, GEO: gene expression omnibus, GLRA3: glycine receptor alpha 3, GNAI3: g protein subunit alpha i3, GO: gene ontology, HDAC1: histone deacetylase 1, hERG: human ether-à-go-go-related gene, HIA: human intestinal absorption, HIF1: hypoxia-inducible factor-1, HRMEC: human retinal microvascular endothelial cells, HSP90AA1: heat shock protein 90 alpha family class a member 1, HSPA8: heat shock protein family a (hsp70) member 8, IL1: interleukin 1, IL6: interleukin 6, KCNE2: potassium voltage-gated channel subfamily e regulatory subunit 2, KCNJ16: potassium voltage-gated channel subfamily j member 16, Keap1: kelch-like ech-associated protein 1, KEGG: the kyoto encyclopaedia of genes and genomes pathway, KRAS: kirsten rat sarcoma viral oncogene homolog, LC3: microtubule-associated protein 1 light chain 3, LD_50_: lethal dose 50%, LGA: lamarckian genetic algorithm, Log Kp: logarithm (base 10) of the skin permeability coefficient (Kp), LPAR2: lysophosphatidic acid receptor 2, MAPK14: mitogen-activated protein kinase 14, MAPK8: mitogen-activated protein kinase 8, MDCK: madin-darby canine kidney cells, MMP9: matrix metallopeptidase 9, mTOR: mammalian target of rapamycin, NaCl: sodium chloride, NEK6/9: nima kinase 6/9, NFE2L2: nuclear factor, erythroid 2 like 2, NFκB: nuclear factor kappa-light-chain-enhancer of activated b cells, NOS3: nitric oxide synthase 3, NPT: the number of particles, pressure, and temperature kept constant, Nrf2: nuclear factor erythroid 2 [NF-E2]-related factor 2, NVT: the number of particles, volume of the system, and the temperature kept constant, p70 S6K: protein 70 S6kinase, PCA: principal component analysis, PI3K: phosphatidylinositol-3kinase, pIC50: negative logarithm (base 10) of the half-maximal inhibitory concentration value, PI3K/AKT: phosphatidylinositol 3-kinase/protein kinase B, PIK3R1: phosphoinositide-3-kinase regulatory subunit 1, pKi: negative logarithm (base 10) of the inhibition constant (Ki), PKM: pyruvate kinase M1/2, PPAR-α: peroxisome proliferator-activated receptor alpha, PPB: plasma protein binding, PPI: protein to protein interaction, PRSS3: serine protease 3, PSA: polar surface area, PTGES: prostaglandin e synthase, PTGS2: prostaglandin-endoperoxide synthase 2, RAGE: receptor for advance glycation endproducts, Rap1: rhoptry-associated protein 1, RAR: retinoic acid receptor, RELA: p65 subunit of nf-κb, Rg: radius of gyration, ROS: reactive oxidative species, RLN3: relaxin 3, RMSD: root mean square deviation, RMSF: root mean square fluctuation, RXR-α: retinoid x receptor alpha, SASA: solvent-accessible surface area, SCTR: secretin receptor, SLC13: solute carrier family 13, SPARC: secreted protein acidic and cysteine rich, SRC: proto-oncogene tyrosine-protein kinase src, T2DM: type 2 diabetes mellitus, TBHQ: tert-butylhydroquinone, TGBG: 1,2,4,6-tetra-o-galloyl beta d glucose, THRB: thyroid hormone receptor beta, TNF: tumor necrosis factor, TP53: tumor protein p53, TPM3: tropomyosin 3, TUBB4B: tubulin beta 4b class ivb, UBB: ubiquitin b, UBC: ubiquitin c, UBR4: ubiquitin protein ligase e3 component n-recognin 4, VEGFA: vascular endothelial growth factor receptor, YWHAB: tyrosine 3-monooxygenase/tryptophan 5-monooxygenase activation protein beta, YWHAQ: tyrosine 3-monooxygenase/tryptophan 5-monooxygenase activation protein theta, α-CR: alpha-crystallin

Amino acids- A: alanine, C: cysteine, D: aspartic acid, E: glutamic acid, F: phenylalanine, G: glycine, H: histidine, I: isoleucine, K: lysine, L: leucine, M: methionine, N: asparagine, P: proline, Q: glutamine, R: arginine, S: serine, T: threonine, V: valine, W: tryptophan, Y: tyrosine

ns: nanoseconds, ps: picoseconds

In molecular docking, the binding orientation and affinity of drug-protein complexes provide insight into how amino acids contribute to the static interaction of drugs to protein active sites for its functional activation. Molecular docking is also useful in screening for druggable hits and optimization of a lead compound with improved affinity and selectivity towards a specific protein with network pharmacology [191]. It has been used to explore new therapeutic avenues for glaucoma [225].

For retinopathy, Julius et al. predicted that several phytochemicals have higher binding affinity for aldose reductase 2 (ALR2) than a reference aldose reductase inhibitor, epalrestat, with a glide score of -7.641 Kcal/mol [206]. From the 14 phytochemicals tested, the half-maximal inhibitory concentration (IC50) was 22.4 nM and 27.3 nM for agnuside and eupalitin-3-O-galactoside against ALR2, respectively, compared to epalrestat with an IC50 98 nM on ARPE-19 cells. This showed that the phytochemicals at lower IC50 concentrations had higher potency for ALR2 targets in retinopathy treatment. In another study, terchebulin, punicalagin, and chebulagic acid from different medicinal plants had superior binding energy towards C4 proteins associated with inflammation-induced retinopathy compared to the reference drug ruboxistaurin [226]. The same phytochemicals showed high binding affinity with VEGFR2 proteins by conventional hydrogen bonds, pi-alkyl, and pi-cation interactions, which was further confirmed for stability through dynamic simulation [227]. This suggested that phytochemicals may offer promising leads for further development with different retinopathy targets and cellular pathways.

Free energy calculation is another method used for quantitative measures of drug-protein complex affinity in a virtual solvent-like environment, crucial for mimicking biological and chemical processes. It is performed using different techniques including alchemical free energy (i.e. free energy perturbation; FEP, thermodynamic integration; TI, and bennett acceptance ratio; BAR), end-point (i.e. molecular mechanics with poisson-boltzmann solvation model; MM/PBSA, and molecular mechanics with generalized born solvation model; MM/GBSA) and enhanced sampling (i.e. metadynamics and umbrella sampling) which are more computationally exhaustive than molecular docking [228].

From a dynamic simulation point of view, the conformational changes between drug-protein and the stability over a time limit can be known for complex interactions at the molecular level [191]. This approach has been performed in drug development for retinopathy treatments by measuring root mean square deviation (RMSD), root mean square fluctuation (RMSF), radius of gyration (Rg) and solvent-accessible surface area (SASA). For example, the phytochemicals apigenin, maritinone, isozeylanone, scopoletin, and scopolin exhibit high interactions with epidermal growth factor receptor (EGFR), proto-oncogene tyrosine-protein kinase src (SRC), akt serine/threonine kinase 1 (AKT1), heat shock protein 90 alpha family class a member 1 (HSP90AA1), and signal transducer and activator of transcription 3 (STAT3) from GeneCards dataset for retinopathy based on network pharmacology [193]. But among this, the interaction between apigenin and SRC complex, with isozeylanone and HSP90AA1 complex have structural stability from 5 ns to the end of the 200 ns simulation time limit. Conversely, the root mean square deviation (RMSD) of apigenin-SRC complex was 0.07 nm, compared to the un-complexed SRC with 0.18 nm. Similarly, isozeylanone-HSP90AA1 complex showed a RMSD of 0.12 nm, compared to the un-complexed HSP90AA1 with 0.15 nm. This indicated that the lower RMSD < 0.30 nm had better stability which were desirable indicators for good drug-protein interactions [229]. In addition, there was minimal conformational changes otherwise known as RMSF in the amino acids’ residue of un-complexed SRC and HSP90AA1 between 0.05 and 0.38 nm, and this similar RMSF range was observed in apigenin-SRC complexes and isozeylanone-HSP90AA1 complexes with the formation of stable intramolecular hydrogen bonds to this effect around the active site loop [193]. Because the RMSF threshold was below 0.5 nm in the un-complexed proteins and phytochemical-protein complex, the outcome was considered a useful indicator for drug-protein interaction stability and rigidity during the simulation time [230]. Additionally, MM/GBSA was used to further assess the binding strength of apigenin-SRC and isozeylanone-HSP90AA1 complexes. The results of both complexes had low binding free energy of − 49.85 kcal/mol and − 45.58 kcal/mol for isozeylanone-HSP90AA1 and apigenin-SRC complex, respectively, which fell below the − 10 kcal/mol range, recognised to provide satisfactory drug-protein interactions, however this range could be influenced by intramolecular bond, and solvation mimicking biological systems [231]. These computational methods by molecular docking, dynamic simulation, and MM/GBSA were complementary with binding affinity and stability; thus, highlighting the possibility of predictions of phytochemical function on proteins involved in retinopathy. Aside from drug-protein conformational changes and stability, the benefit of dynamic simulations can estimate the dissolution and solubility of drugs in the delivery system of polymers [232].

Pharmacokinetics (PK) is the science of drug behaviour in the body from administration to elimination over time and is another assessment useful for retinopathy treatment investigation. Earlier studies demonstrated PK simulation with different numerical modelling such as non-compartmental PK, classical compartmental PK, physiological based PK, mixed effects based PK and computational fluid dynamics (CFD) [233]. All the modelling were based on the complexity of the anatomy and physiological models used for drug exposure [233]. Some commercial software and online databases use information built into the system to mimic anatomical and physiological behaviour of organs for various drug classes to provide numerical estimations for topical dosing and intravitreal injection formulations [234]. This then predicted absorption, distribution, metabolism, excretion, and toxicity (ADMET) properties useful to guide experimental investigations. For example, two clinical studies used PK simulation to estimate the clearance of anti-VEGF using 1.25 mg bevacizumab and 0.50 mg ranibizumab in the aqueous and vitreous humor of patients with macular edema secondary to AMD, retinopathy, and retinal vein occlusion. The PK simulation predicted the rate of clearance to be within 7 days, which was slightly shorter than the experimental clearance of 9 days for the bevacizumab administered via intravitreal injection [235]. This inaccuracy was repeated for ranibizumab, with the predicted rate of clearance within 7 days that was experimentally cleared within 3 days from the aqueous and vitreous humor [236]. The large molecular weight of bevacizumab (149 kDa) contributed to its long-term clearance, compared to the low weight of ranibizumab (48 kDa). These differences can be useful to improve the dosing of ranibizumab and bevacizumab for retinopathy treatments. In another study [237], IgG1 Fab, an anti-VEGF agent from an episcleral implant was computationally predicted to take 8 weeks for the sustained delivery to the back of the eye, which was the same as clinical intravitreal injections. Therefore, PK simulation can provide predictions useful to guide experimentation and clinical practice to reduce injection-related risks to the eye [237].

Another capability of PK simulation was associated with the physicochemical properties of compounds. These properties consider the “Lipinski rule of five” which use molecular weight, lipophilicity, hydrogen bond donor, hydrogen bond acceptor, and does not violate more than one of the four earlier criteria. Drugs or bioactive compounds that satisfy these rules were more likely absorbed orally (oral bioavailability), and with drug-likeness attributes. This suggested that bioactive compounds can be optimized for drug design and development by medicinal chemistry against human diseases [234]. Compounds or phytochemicals that satisfy the Lipinski rule of five were drug-like and orally bioavailable using the threshold of ≥ 0.18 and ≥ 30%, respectively. For example, numerous phytochemicals from Asian herbal plants pass the drug-likeness and oral bioavailability threshold, and were identified as retinopathy protein targets including *Cichorium intybus* (24 phytochemicals) [9], *R. salviae* (65 phytochemicals) [200], and combinations of *Plumbago zeylanica* and *Solanum xanthocarpum* (28 phytochemicals) [193], *Cuscuta semen*, *Plantaginis semen*, and *R. rehmanniae* (42 phytochemicals) [214], *Erigeron breviscapus*, *Astragalus membranaceus*, and *Radix puerariae* (28 phytochemicals) [199], *Cistanche*, *Lucid ganoderma*, *Lycium barbarum*, *Angelica sinensis*, *Semen Cuscutae*, *Rhizoma atractylodis*, *Ligusticum wallichii*, *Salvia miltiorrhiza* and *Codonopsis pilosula* (227 phytochemicals) [196], *Panax notoginseng*, *Astragalus membranaceus*, *Salvia miltiorrhiza*, and *Scrophularia ningpoensis* (102 phytochemicals) [202] and *Ginkgo biloba* (27 phytochemicals) [223]. Among the phytochemicals, eupalitin-3-O-galactoside, picroside II, agnuside, 7-O-methylwogonin [206] magniferin [205], ferulic acid, apigenin, luteolin-7-O-glucoside, luteolin, chrysosplenol, and kaempferol [9], have been validated by in-vitro analysis for retinopathy treatment.

In addition, these computational methods have been helpful in highlighting the benefits of flavonoids from *Ginkgo biloba* confirmed in experimental and clinical trial interventions as a retinopathy therapy [223, 238]. There were computational predictions that linked macular carotenoids against diseases like diabetes [204, 239], photoaging [240], obesity [241], gut disorders [242], and cancer [243], with experimental validation. However, there were no comprehensive computational investigations to link macular carotenoids (LMZ) to specific retinopathy protein target except a molecular docking investigation which provide only limited information [221]. Although literature of the computational approaches for retinopathy is still in the early stage of development, the understanding from the investigations so far were able to provide new hints on how bioactive compounds interact with protein target links to retinopathy-specific pathways in a virtual ocular model and this can be beneficial against pathways associated with retinopathy.

## Conclusion

Computational, experimental and clinical studies highlight the benefits of herbal-based compounds for early retinopathy remedy, with minimal side effects, low cost and wide accessibility as supplements or through the diet, particularly against oxidative stress, inflammation, apoptosis, angiogenesis, and visual function deficits. Specifically, polyphenols and carotenoids were the main bioactive compounds useful in early retinopathy treatments, the bioaccumulation of macular carotenoids (specifically lutein and zeaxanthin; 80–90% of the total carotenoids) in the retina and macular after ingestion of carotenoid-rich foods or supplements have been associated with benefits in eye functions, suggesting its importance in overall eye health and maintenance. Furthermore, advancements in polymers like hydrogel formulations and encapsulations have created a foundation for diverse applications, enabling the delivery of novel medicines for conditions beyond retinopathy and potentially addressing more challenging diseases.

While herbal supplementation shows promise for disease prevention, its translational application for treating established diseases is limited, even at early stages. The slow onset of action, high inter-individual variability in bioavailability, and dependence on dietary/lifestyle factors make it unsuitable as a primary therapeutic intervention [244]. Instead, its strongest translational value lies in long-term preventive strategies within public health and nutritional programs targeting at-risk populations.

## Data Availability

No datasets were generated or analysed during the current study.
